# Automated Neuron Reconstruction from 3D Fluorescence Microscopy Images Using Sequential Monte Carlo Estimation

**DOI:** 10.1007/s12021-018-9407-8

**Published:** 2018-12-12

**Authors:** Miroslav Radojević, Erik Meijering

**Affiliations:** Biomedical Imaging Group Rotterdam, Departments of Medical Informatics and Radiology, Erasmus University Medical Center, Rotterdam, The Netherlands

**Keywords:** Neuron reconstruction, Bayesian filtering, Sequential Monte Carlo estimation, Particle filtering, Fluorescence microscopy

## Abstract

Microscopic images of neuronal cells provide essential structural information about the key constituents of the brain and form the basis of many neuroscientific studies. Computational analyses of the morphological properties of the captured neurons require first converting the structural information into digital tree-like reconstructions. Many dedicated computational methods and corresponding software tools have been and are continuously being developed with the aim to automate this step while achieving human-comparable reconstruction accuracy. This pursuit is hampered by the immense diversity and intricacy of neuronal morphologies as well as the often low quality and ambiguity of the images. Here we present a novel method we developed in an effort to improve the robustness of digital reconstruction against these complicating factors. The method is based on probabilistic filtering by sequential Monte Carlo estimation and uses prediction and update models designed specifically for tracing neuronal branches in microscopic image stacks. Moreover, it uses multiple probabilistic traces to arrive at a more robust, ensemble reconstruction. The proposed method was evaluated on fluorescence microscopy image stacks of single neurons and dense neuronal networks with expert manual annotations serving as the gold standard, as well as on synthetic images with known ground truth. The results indicate that our method performs well under varying experimental conditions and compares favorably to state-of-the-art alternative methods.

**Electronic supplementary material:**

The online version of this article (10.1007/s12021-018-9407-8) contains supplementary material, which is available to authorized users.

## Introduction

The brain is regarded as one of the most complex and enigmatic biological structures. Composed of an intricate network of tree-shaped neuronal cells (Ascoli 2015), together forming a powerful information processing unit, it performs a myriad of functions that are essential to living organisms (Kandel et al. 2012). Obtaining a blue print of the architecture of this network, including the morphologies and interconnectivities of the neurons in various subunits, helps to understand how the brain works (Ascoli 2002; Donohue and Ascoli 2008; Cuntz et al. 2010), including how neurodegenerative disease processes alter its function. A key instrument in this endeavor is microscopic imaging, as it allows detailed visualization of neuronal cells in isolation and in tissue, thus providing the means to study their structural properties quantitatively (Senft 2011).

Quantitative measurement and statistical analysis of neuronal cell and network properties from microscopic data rely on the ability to obtain accurate digital reconstructions of the branching structures (Halavi et al. 2012) in the form of a directional tree of connected nodes (Ascoli et al. 2007). The ever increasing amount of available image data calls for automated computational methods and software tools for this purpose, as manual delineation of neurons is extremely cumbersome even in single image stacks, and is downright infeasible in processing large numbers of images (Svoboda 2011; Senft 2011). Automating neuron reconstruction requires solving fundamental computer vision problems such as detecting and segmenting tree-like image structures (Meijering 2010; Donohue and Ascoli 2011; Acciai et al. 2016). This is complicated by the large diversity of neuron types, imperfections in cell staining, optical distortions, inevitable image noise, and other causes of ambiguity in the image data. Consequently, with the current state-of-the-art, manual proof-editing of automatically obtained digital reconstructions is often necessary (Peng et al. 2011b). Recent international initiatives such as the DIADEM challenge (Gillette et al. 2011) and the BigNeuron project (Peng et al. 2015a, 2015b) have catalyzed research in automated neuron reconstruction but have also clearly revealed that further improvement is still very much needed before computers can fully replace manual labor in performing this task.

With this paper we aim to contribute to the developments in the field by proposing a novel fully automated neuron reconstruction method based on probabilistic filtering techniques. Starting from seed points that have a high probability of being centered at neuronal branches, our method recursively traces these branches by sequential Monte Carlo estimation, using state transition and measurement models designed specifically for this purpose. This results in a series of possibly overlapping but probabilistically independent estimates of the branches, which are subsequently combined into a refined estimate of the actual branch centerlines using mean-shifting. We presented early versions of the method at conferences (Radojević et al. 2015; Radojević and Meijering 2017b) and donated one implementation of it (named Advantra) for inclusion in the BigNeuron benchmarking study (Peng et al. 2015a, 2015b). Since then we have improved the method and its software implementation and have significantly extended its experimental evaluation. Here we provide a detailed description of the method, its implementation, and the experimental results, and show that it performs favorably compared to several state-of-the-art neuron reconstruction methods from the BigNeuron project as well as an alternative probabilistic method (Radojević and Meijering 2017a). The source code of our software implementation will be released along with this paper.

**Fig. 1:**
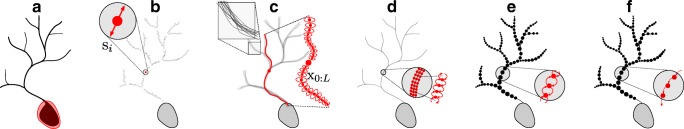
Schematic overview of the six main steps of the proposed method: **a** soma extraction, **b** seed extraction, **c** branch tracing, **d** trace refinement, **e** node grouping, **f** tree construction

## Related Work

Early methods and tools for digital neuron reconstruction were semi-automatic and required extensive manual intervention for their initialization and operation or the curation of faulty results (Glaser and Van der Loos 1965; Capowski and Sedivec 1981; Glaser and Glaser 1990; Masseroli et al. 1993). With the increasing capabilities of computers it became possible to store and process 3D images of neurons (Cohen et al. 1994; Belichenko and Dahlström 1995). More recently, the state-of-the-art in the field has moved towards full automation of neuron reconstruction, and various freely available software tools are now available for this purpose (Peng et al. 2010; Longair et al. 2011; Peng et al.2014a, 2014a), though the need for flexible editing tools has remained unabated (Luisi et al. 2011; Dercksen et al. 2014).

Neuron reconstruction methods typically have a modular design where each module or stage of the processing pipeline deals with different structural objects. Depending on the subproblems being solved, modules can operate independently, or work together for example to combine local and global processing, possibly requiring multiple iterations. Several subproblems that can be identified in the literature include image prefiltering and segmentation (Zhou et al. 2015; Türetken et al. 2011; Sironi et al. 2016; Mukherjee and Acton 2013), soma (cell body) detection and segmentation (Quan et al. 2013), landmark points extraction (Al-Kofahi et al. 2008; Wang et al. 2011; Choromanska et al. 2012; Baboiu and Hamarneh 2012; Su et al. 2012; Radojević et al. 2016), neuron arbor tracing (Zhao et al. 2011; Liu et al. 2016; Leandro et al. 2009; Radojević and Meijering 2017a; Xiao and Peng 2013), and assembling the final tree-like graph structure (Zhou et al. 2016; Türetken et al. 2011; Yuan et al. 2009). In the remainder of this section we briefly review techniques for solving each of these subproblems. Since our primary goal in this paper is to present a new method, the review is not meant to be exhaustive, but to put our method into context.

The pool of neuron reconstruction methods is very diverse (Meijering 2010; Donohue and Ascoli 2011; Acciai et al. 2016; Peng et al. 2015a) but there are also many commonalities. For example, image prefiltering to enhance tubular structures is typically carried out using Hessian or Jacobian based processing (Xiong et al. 2006; Al-Kofahi et al. 2008; Yuan et al. 2009; Wang et al. 2011). And to cope with uneven staining, adaptive thresholding (Zhou et al. 2015), perceptual grouping (Narayanaswamy et al. 2011), and vector field convolution (Mukherjee et al. 2015) have been used. For image segmentation (separating foreground from background), a wide variety of methods has been proposed, including the use of feature-based classifiers (Türetken et al. 2011; Chen et al. 2015; Jiménez et al. 2015), tubularity based supervised regression (Sironi et al. 2016), and even deep learning (Li et al. 2017). The general difficulty of supervised methods, however, is their need for extensive manual annotation for training to arrive at usable segmentation models. In our proposed method we have chosen to avoid this by using carefully designed explicit models.

For the detection and segmentation of the neuronal somas, which typically have a much larger diameter than the dendritic and axonal branches, a simple and efficient solution is to apply morphological closing and adaptive thresholding (Yan et al. 2013). An alternative is to use shape fitting approaches (Quan et al. 2013). Next, to initialize and/or guide the segmentation of the arbor, landmark points are often extracted using image filters that specifically enhance tubular structures (Wang et al. 2011; Türetken et al. 2011; Choromanska et al. 2012; Su et al. 2012; Radojević et al. 2016), a popular one being the so-called “vesselness filter” (Frangi et al. 1998). In our proposed method we have adopted classical approaches for soma and seed point detection as detailed in the next section.

Segmentation or tracing of all branches of the dendritic and axonal trees is the main challenge of the reconstruction problem. A widely used approach to overcome the difficulties caused by imperfect staining and image noise is to use techniques that find globally optimal paths between seed points by minimizing a predefined cost function (Meijering et al. 2004; Peng et al. 2011a; Longair et al. 2011; Quan et al. 2016). But many other concepts have been proposed as well, including model fitting (Schmitt et al. 2004; Zhao et al. 2011), contour extraction (Leandro et al. 2009), active contour segmentation (Wang et al. 2011; Luo et al. 2015), level-set or fast-marching approaches (Xiao and Peng 2013; Basu and Racoceanu 2014), path-pruning from oversegmentation (Peng et al. 2011a), distance field tracing (Yang et al. 2013), marching rayburst sampling (Ming et al. 2013), marked point processing (Basu et al. 2016), iterative back-tracking (Liu et al. 2016), and learning based approaches (Chen et al. 2015; Gala et al. 2014; Santamaría-Pang et al. 2015). In recent works we have shown the great potential of probabilistic approaches to neuron tracing (Radojević et al. 2015; Radojević and Meijering 2017a, 2017a) which formed the basis for the new fully automated neuron reconstruction method presented and evaluated in the next sections.

The final aspect of neuron reconstruction is the assembling of the complete neuronal tree structure from possibly many partial or overlapping traces and putting it into a format that is both representative and suitable for further automated analysis. This is typically solved by graph optimization strategies such as the minimum spanning tree (MST), the alternative K-MST (Türetken et al. 2011; González et al. 2010), or integer programming (Türetken et al. 2013). To deal with very large data sets it has also been proposed to assemble the 3D graph representation through tracing in 2D projections and applying reverse mapping (Zhou et al. 2016). However, with the advent of sophisticated assemblers such as UltraTracer (Peng et al. 2017), it is possible to extend any base tracing algorithm to deal with arbitrarily large volumes of neuronal image data (Peng et al. 2017). Therefore, in our proposed method, we do not use projections but perform the tracing in the original image (sub)volumes. And to obtain the graph representation we propose a new approach to refining and grouping the individual traces.

## Proposed Method

The pipeline of our proposed method consists of six steps (Fig. 1) described in detail in the following subsections. We assume that image stacks contain a single neuron (one soma) or just an arbor (no soma) as in the DIADEM (Brown et al. 2011) and BigNeuron data (Peng et al. 2015a). In short, we first extract the soma and a set of seeds, which serve to initialize our probabilistic branch tracing scheme. The resulting traces are iteratively refined and their corresponding nodes spatially grouped into a representative node set that is traversed to form the final reconstruction.

### Soma Extraction

The soma typically has a considerably larger diameter than the individual branches of the neuronal arbor (Fig. 1a). Thus it can be easily extracted using morphological filtering operations (Yan et al. 2013). Specifically, in our method, we apply grayscale erosion to remove all branches and leave only the (eroded) soma. To this end, the radius *r*_*s*_ of the structuring element needs to be larger than the largest expected branch radius in a given data set, and smaller than the expected soma radius. The resulting image is then smoothed using a Gaussian filter with standard deviation equal to *r*_*s*_ and segmented using max-entropy thresholding (Radojević et al. 2016) to obtain a blob corresponding to the soma. For computational efficiency both the erosion and the Gaussian smoothing operation are carried out by separable filtering. In this paper we model the soma in the final graph representation of the neuron as a single spherical node with position equal to the centroid of the segmented blob and radius equal to the average distance of the blob voxels to the centroid. Alternatively, we could model the soma with a set of nodes that together represent the blob as accurately as we like, but in our applications this is not needed.

**Fig. 2:**
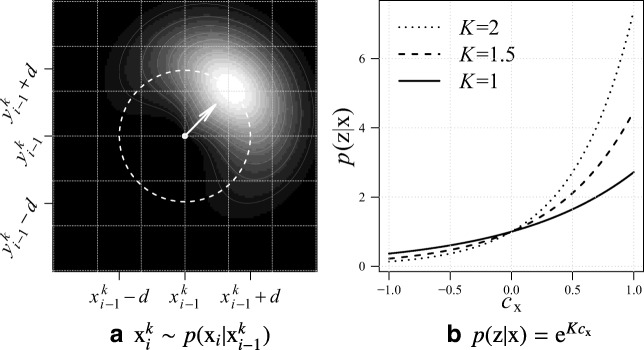
Functions used in the prediction and update steps of the SMC filtering: **a** the prediction importance sampling distribution (for ease of visualization a 2D example is given) and **b** the measurement likelihood function for different values of *K*. Reprinted with permission from Radojević and Meijering (2017b)

### Seed Extraction

To initialize the branch tracing we extract a set of seed points (Fig. 1b). These seeds are points with very high likelihood of being centered on a branch. In our method we estimate this likelihood using a Hessian-based multiscale tubularity filter (Frangi et al. 1998).^1^ It computes for every voxel in an image *I* the Gaussian-smoothed second-order derivatives:

1 $$ \mathcal{H}_{\sigma} = \left[\begin{array}{ccc} \mathcal{D}_{xx} & \mathcal{D}_{xy} & \mathcal{D}_{xz} \\ \mathcal{D}_{yx} & \mathcal{D}_{yy} & \mathcal{D}_{yz} \\ \mathcal{D}_{zx} & \mathcal{D}_{zy} & \mathcal{D}_{zz} \end{array}\right] = \left[\begin{array}{ccc} \frac{\partial^{2}}{(\partial x)^{2}} & \frac{\partial^{2}}{\partial x \partial y} & \frac{\partial^{2}}{\partial x \partial z} \\[1ex] \frac{\partial^{2}}{\partial y \partial x} & \frac{\partial^{2}}{(\partial y)^{2}} & \frac{\partial^{2}}{\partial y \partial z} \\[1ex] \frac{\partial^{2}}{\partial z \partial x} & \frac{\partial^{2}}{\partial z \partial y} & \frac{\partial^{2}}{(\partial z)^{2}} \end{array}\right] (I \ast G_{\sigma}) $$

where ∗ denotes convolution, *G*_*σ*_ the Gaussian filter at scale *σ*, and $\mathcal {H}_{\sigma }$ the resulting local Hessian matrix at that scale. The eigenvalues |*λ*_1_|≤|*λ*_2_|≤|*λ*_3_| of $\mathcal {H}_{\sigma }$ are indicative of the geometry of the local image structure and are used to quantify its tubularity as (Frangi et al. 1998):

2 $$ \upsilon = \left\{\begin{array}{ll} 0 & \text{if } \lambda_{2}>0 \text{ or } \lambda_{3}>0 \\ \left( 1-\text{e}^{-\frac{\mathcal{R}_{a}^{2}}{2\alpha^{2}}}\right) \text{e}^{-\frac{\mathcal{R}_{b}^{2}}{2\beta^{2}}} \left( 1 - \text{e}^{-\frac{\mathcal{S}^{2}}{2c^{2}}}\right) & \text{otherwise} \end{array}\right.  $$

with the free parameters typically set to *α* = *β* = 0.5 and *c* to half the maximum Hessian norm and where

3 $$ \mathcal{R}_{a} = \frac{|\lambda_{2}|}{|\lambda_{3}|} \qquad \mathcal{R}_{b} = \frac{|\lambda_{1}|}{\sqrt{|\lambda_{2} \lambda_{3}|}} \qquad \mathcal{S} = \sqrt{{\lambda_{1}^{2}} + {\lambda_{2}^{2}}}  $$

For each voxel location p = [*x*,*y*,*z*] the spatial scale of the local image structure is taken to be the Gaussian *σ* at which the filter yields the highest tubularity value *υ*. And the orientation of the structure is then taken to be the eigenvector v = [*v*_*x*_,*v*_*y*_,*v*_*z*_] corresponding to the smallest absolute eigenvalue *λ*_1_ of the Hessian matrix $\mathcal {H}_{\sigma }$.

From the resulting tubularity map, initial seed points s_*i*_ = [p_*i*_,v_*i*_,*σ*_*i*_] are selected whose tubularity value is the highest in a cylindrical neighborhood with radius 3*σ*_*i*_ and length *σ*_*i*_, centered at p_*i*_, and oriented along v_*i*_. For this purpose we use a find-maxima function ported from ImageJ, which applies a noise tolerance *τ* to prune insignificant local maxima (Ferreira and Rasband 2012). The final set of seeds is subsequently obtained by excluding the maxima where the correlation of the image with a cylindrical template model is too low, using exactly the same criterion as for termination of branch tracing, described next.

### Branch Tracing

For each seed s_*i*_, our method traces the local image structure in two directions, + v_*i*_ and −v_*i*_, producing a pair of local traces (Fig. 1c). A trace is considered to consist of a sequence of hidden states, $\mathrm {x}_{0:L} = (\mathrm {x}_{0},\dots ,\mathrm {x}_{L})$, where x_0_ is the initial state extrapolated from the seed *s*_*i*_, and x_*L*_ is the last state of the trace. Similar to the seeds, the states $\mathrm {x}_{i} = \left [ \mathrm {p}_{i}, \mathrm {v}_{i}, \sigma _{i} \right ]$ contain estimates of the position $\mathrm {p}_{i} = \left [ x_{i}, y_{i}, z_{i} \right ]$, the direction $\mathrm {v}_{i} = \left [ v_{x_{i}}, v_{y_{i}}, v_{z_{i}} \right ]$, and the scale *σ*_*i*_ of the underlying neuron branch. The states are estimated sequentially in a probabilistic fashion using Bayes’ rule:

4 $$ p(\mathrm{x}_{i} | \mathrm{z}_{0:i}) \propto p(\mathrm{z}_{i} | \mathrm{x}_{i}) \!\!\int\!\! p(\mathrm{x}_{i} | \mathrm{x}_{i-1}) p(\mathrm{x}_{i-1} | \mathrm{z}_{0:i-1}) \text{dx}_{i-1}  $$

where *p*(x_*i*_|z_0:*i*_) is the posterior probability distribution of the state x_*i*_ given measurements z_0:*i*_ from the first to the current iteration, *p*(x_*i*_|x_*i*− 1_) is the state transition prior, and *p*(z_*i*_|x_*i*_) is the likelihood of measuring z_*i*_ given state x_*i*_. It is assumed that the state transition is a Markovian process and the measurements are independent. To allow for nonlinearities in the process, we solve the estimation problem (4) using sequential Monte Carlo (SMC) filtering (Doucet et al. 2001), also known as particle filtering (Arulampalam et al. 2002). Here the posterior is approximated using a set of *N* samples $\mathrm {x}_{i}^{k}$ with corresponding weights ${w_{i}^{k}}$ as:

5 $$ p(\mathrm{x}_{i} | \mathrm{z}_{0:i}) \approx {\sum}_{k = 1}^{N} {w_{i}^{k}} \delta(\mathrm{x}_{i} - \mathrm{x}_{i}^{k})  $$

where the weights are normalized so that ${\sum }_{k} {w_{i}^{k}} = 1$ and

6 $$ \delta(\mathrm{x}) = \left\{\begin{array}{ll} \infty & \text{if } \mathrm{x} = 0\\[0.5ex] 0 & \text{otherwise} \end{array}\right. \quad\text{with}\quad{\int}_{-\infty}^{\infty}\!\!\!\!\delta(\mathrm{x})\text{dx} = 1 $$

Each iteration in SMC filtering consists of a prediction step and an update step. In the prediction step, given the samples $\mathrm {x}_{i-1}^{k}$ from the previous iteration, *N* new samples $\mathrm {x}_{i}^{k}$ are drawn using the state transition prior. The importance sampling distribution that we use for this is (Fig. 2a):

7 $$ p(\mathrm{x}_{i} | \mathrm{x}_{i-1}^{k}) = \left\{\begin{array}{l} \displaystyle\frac{\mathrm{e}^{\kappa\mathrm{v}_{i} \cdot \mathrm{v}_{i-1}^{k} -\ \frac{(d_{i}-d)^{2}}{2 (d/3)^{2}} -\ \frac{(\sigma_{i}-\sigma_{i-1}^{k})^{2}}{2\zeta^{2}}}}{2 \pi I_{0}(\kappa)\eta} \\[2ex] \begin{array}{ll} & \qquad\text{for \(d_{i} \leq 2d \land \sigma_{i} \leq 3\zeta\)}{ }\\ 0 & \qquad\text{otherwise} \end{array} \end{array}\right.  $$

where *I*_0_ denotes the zero-order Bessel function of the first kind, *κ* is the circular variance parameter, *η* is a normalization factor that makes the prediction over all *N* samples integrate to unity, $d_{i}= || \mathrm {p}_{i} - \mathrm {p}_{i-1}^{k} ||$ is the Euclidean distance between the predicted position and the sample position in the previous iteration, *d* is the tracing step size, and *ζ* the scale variance parameter. Each predicted state is assigned a unit direction $\mathrm {v}_{i} = (\mathrm {p}_{i} - \mathrm {p}_{i-1}^{k}) / || \mathrm {p}_{i} - \mathrm {p}_{i-1}^{k} ||$ defined by two consecutive positions. And $\sigma _{i}-\sigma _{i-1}^{k}$ represents the difference in scales, which contributes to the importance sampling function by a Gaussian component, giving a higher value to state samples that retain the scale.

**Fig. 3:**
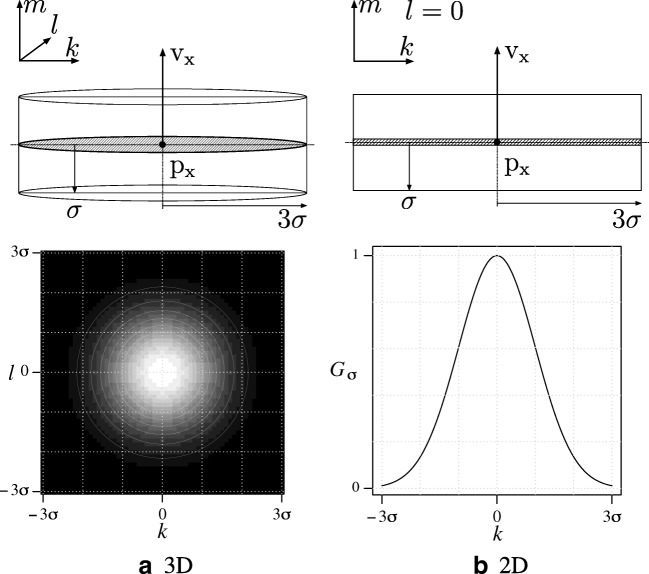
Cylindrical template intensity model *G*_*σ*_. The model has a Gaussian profile in coordinates *k* and *l* and is constant in coordinate *m*. Both the 3D **(a)** and the 2D **(b)** version is shown

In the update step, the newly drawn samples are updated using the following likelihood function (Fig. 2b):

8 $$ p(\mathrm{z} | \mathrm{x}) = e^{K c_{\mathrm{x}}} $$

where *K* determines the sensitivity to the normalized cross-correlation *c*_x_ ∈ [− 1,1], which quantifies the similarity of the underlying image structure for $\mathrm {x}=\left [ \mathrm {p}, \mathrm {v}, \sigma \right ]$ to a cylindrical template model with Gaussian profile (Fig. 3):

9 $$ c_{\mathrm{x}} = \frac{ {\sum}_{k,l,m} \left( I(\mathrm{p}^{\prime})-\bar{I}\right) \left( G_{\sigma}-\bar{G}\right) }{ \!\!\sqrt{ {\sum}_{k,l,m}\left( I(\mathrm{p}^{\prime})-\bar{I}\right)^{2} {\sum}_{k,l,m}\left( G_{\sigma}-\bar{G}\right)^{2}} } $$

10 $$ \mathrm{p}^{\prime} = \mathrm{p}^{\prime}(k,l,m) = \mathrm{p} + k \mathrm{u} + l \mathrm{w} + m \mathrm{v} $$

11 $$ G_{\sigma} = G_{\sigma}(k,l,m)=G_{\sigma}(k,l)=\mathrm{e}^{-\left( k^{2}+l^{2}\right)/2\sigma^{2}} $$

where (*k*,*l*,*m*) are the template coordinates, which transform to $\mathrm {p}^{\prime }$ in image coordinates since the template is centered at p and is oriented in the direction v and has scale *σ* of x, and by definition u ⊥ v, w ⊥ v, and u ⊥ w. The summation is limited to ⌊− 3*σ*⌋≤ *k*,*l* ≤⌈3*σ*⌉ and ⌊*σ*⌋≤ *m* ≤⌈*σ*⌉ which corresponds to the spatial extent of the template. $\bar {I}$ and $\bar {G}$ denote the mean of the image intensities and of the template intensities, respectively, within the mentioned limits. The value of *c*_x_ is independent of intensity scalings and offsets and thus provides us with a robust measure of structural resemblance, which may range from − 1 (inverse correlation), to 0 (no correlation), to + 1 (full correlation). The weights of the samples are updated accordingly as:

12 $$ {w_{i}^{k}} \propto w_{i-1}^{k} p\left( \mathrm{x}_{i}^{k} | \mathrm{x}_{i-1}^{k}\right) \mathrm{e}^{K c_{\mathrm{x}_{i}^{k}}} $$

and renormalized so that ${\sum }_{k} {w_{i}^{k}} = 1$. To avoid weight deterioration, systematic resampling (Kitagawa 1996) is performed each time the effective sample size *N*_eff_ (Kong et al. 1994) falls below 80% of *N*. The final state estimate after each iteration *i*, which constitutes a node of the trace, is computed from the weighted samples as the centroid:

13 $$ \hat{\mathrm{x}}_{i} = \sum\limits_{k} {w_{i}^{k}} \mathrm{x}_{i}^{k} $$

Filtering is terminated if the average correlation value ${\sum }_{k} c_{\mathrm {x}_{i}^{k}}/N$ drops below the threshold $c_{\min }$, indicating the end of the underlying neuron branch in the image, or if the iteration limit *L* is reached. Since the filtering is done for each seed, and in both (opposite) directions, the same neuron branch may be traced many times over, but in a probabilistically independent way, providing accumulating evidence about the presence and location of the branches. However, to avoid excessive over-tracing and to reduce the computation time, we also monitor the node density *D*_*n*_ per image volume unit of *n* voxels and terminate the tracing if the density in the current position exceeds the limit *δ*_*n*_. In principle *n* can be any number, but in our work we typically consider *n* = 1 (single voxel), *n* = 5 (4-connected in 3D), and *n* = 9 (8-connected in 3D), which is sufficient given that the image stacks often have a large voxel anisotropy.

**Fig. 4:**
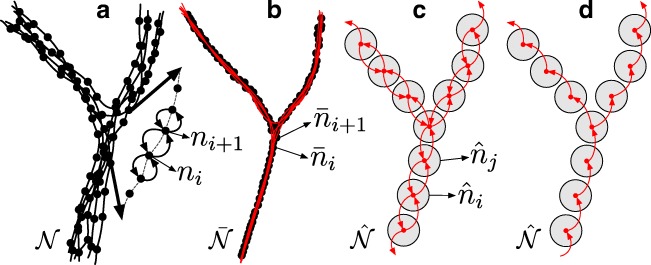
Trace merging: **a** accumulated traces, **b** trace refinement, **c** node grouping, **d** tree traversal

### Trace Refinement

After the tracing step, each neuron branch may have multiple corresponding traces, and each trace node has bidirectional links to neighboring nodes (Figs. 1d and 4a) to allow trace traversal in any of the possible directions in the final tree construction step. Denoting the total number of traces by *T*, and the nodes of any given trace *t* by ${n_{i}^{t}}$, $i = 1,\dots ,M^{t}$, we may write the complete set of nodes as:

14 $$ \mathcal{N} = \left\{ \left\{ {n_{1}^{1}},\dots,n_{M^{1}}^{1}\right\}, \dots, \left\{ {n_{1}^{T}},\dots,n_{M^{T}}^{T}\right\} \right\} $$

but in the sequel we write the elements of $\mathcal {N}$ more generally as *n*_*k*_, $k = 1,\dots ,M$, where $M={\sum }_{t = 1}^{T}M^{t}$. Each node *n*_*k*_ contains an estimate of the center position $\mathrm {p=}\left (x,y,z\right )$ and the cross-sectional radius $\left (r\right )$ of the underlying branch structure, as well as the cross-correlation $\left (c\right )$ with the cylindrical Gaussian template model, and a set $\left (\mathcal {I} \right )$ containing the indices in $\mathcal {N}$ of the neighboring nodes:

15 $$ n_{k} = \lbrace \mathrm{p}_{k}, r_{k}, c_{k}, \mathcal{I}_{k} \rbrace $$

where $\mathcal {I}_{k}$ has either two elements (in the case of a body node) or just one (in the case of a terminal node).

The goal of the trace refinement step is to exploit the cumulative evidence provided by the over-tracing in the previous step to improve the individual node estimates. Specifically, we update each node *n*_*k*_ to:

16 $$ \bar{n}_{k} = \left\lbrace \bar{\mathrm{p}}_{k}, \bar{r}_{k}, \bar{c}_{k}, \bar{\mathcal{I}}_{k} \right\rbrace $$

by applying mean-shifting (Cheng 1995), resulting in an updated node set $\bar {\mathcal {N}}$. Mean-shifting iteratively moves each node element to the local mean of the nodes in its vicinity:

17 $$ \bar{n}_{k} = \frac{{\sum}_{n \in \mathcal{N}} \mathrm{\Psi}(n-n_{k}) \cdot n}{{\sum}_{n \in \mathcal{N}} \mathrm{\Psi}(n-n_{k})} $$

18 $$ \mathrm{\Psi}(n-n_{k}) = \left\{\begin{array}{ll} 1 & \text{if } \| \mathrm{p} - \mathrm{p}_{k} \| \leq r_{k} \\ 0 & \text{otherwise} \end{array}\right. $$

This reduces the variance of the estimates but preserves the linking of the nodes: $\bar {\mathcal {I}}=\mathcal {I}$. In practice, five iterations are sufficient to reach satisfactory radial trace alignment (Fig. 4b). The kernel size used in the mean-shifting process is taken to be the initial radius of each node. In our implementation, prior to mean-shifting, we resample all traces with a step size of one voxel to get a more fine-grained result. 
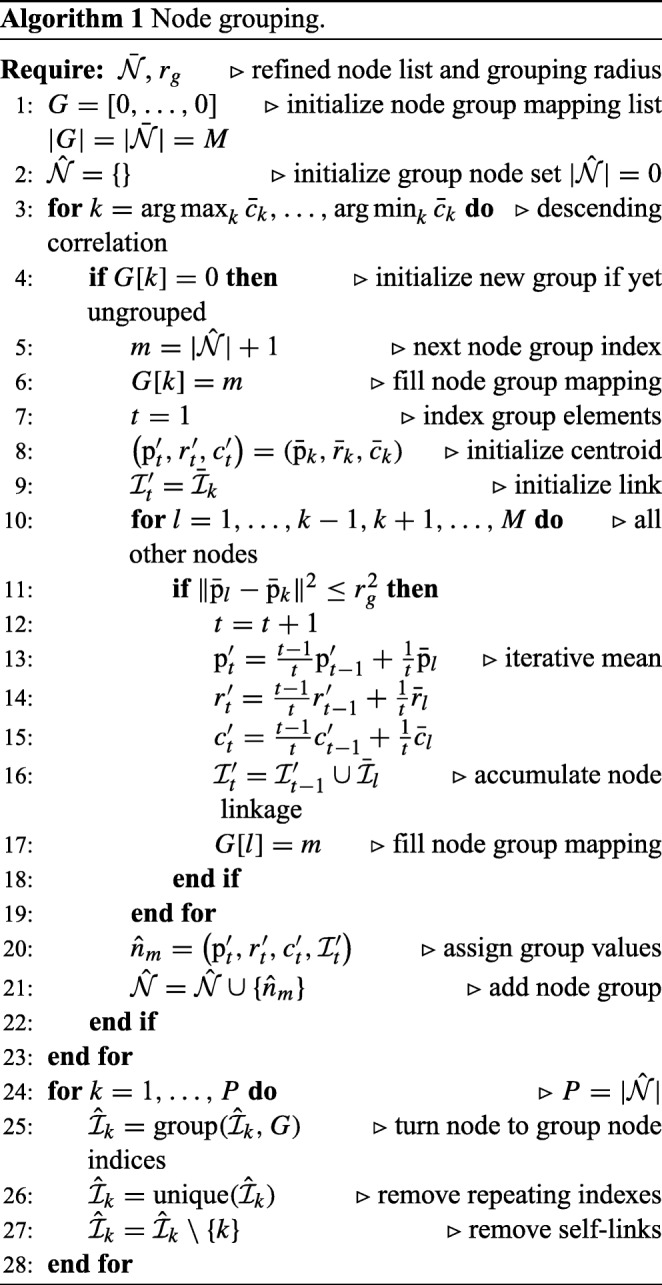


### Node Grouping

Although the previous step results in refined node estimates, it keeps the total number of nodes and corresponding multiple traces. The next step is to merge overlapping traces and obtain a single trace for each neuron branch. In our method this is accomplished by the process of node grouping (Figs. 1e and 4c) as detailed in Algorithm 1. It iteratively takes from the refined set $\bar {\mathcal {N}}$ an as-yet ungrouped node with the highest cross-correlation value, finds all its neighboring nodes within the predefined Euclidean distance *r*_*g*_, and groups them by calculating the mean value of each element. In our implementation we use unweighted averaging for this. Alternatively, weighted averaging could be used, based on the cross-correlation scores. The node links within a group are accumulated and their indexes mapped to the group node index list. This results in a new set $\hat {\mathcal {N}} = \lbrace \hat {n}_{1},\dots ,\hat {n}_{P} \rbrace $, *P* ≤ *M*, of group nodes:

19 $$ \hat{n}_{k}=\left\lbrace\hat{\mathrm{p}}_{k}, \hat{r}_{k}, \hat{c}_{k}, \hat{\mathcal{I}}_{k}\right\rbrace $$

20 $$ \hat{n}_{k} = \frac{ {\sum}_{n \in \bar{\mathcal{N}}} \mathrm{\Psi}(n - \bar{n}_{k}) \cdot n }{ {\sum}_{n \in \bar{\mathcal{N}}} \mathrm{\Psi}(n - \bar{n}_{k}) } $$

and any two $\hat {n}_{i}$ and $\hat {n}_{j}$ are connected if there exists a link between any of the refined nodes captured by these two, as revealed by the accumulated index sets $\hat {\mathcal {I}}_{i}$ and $\hat {\mathcal {I}}_{j}$. Thus, all existing inter-node connections $\bar {\mathcal {I}}$ are preserved, and are projected into the inter-group connections $\hat {\mathcal {I}}$ (see Supplementary Fig. ?? for an example case).

**Table 1:** Parameters of our method and their default values and grid search values used for each data set in the experiments

Parameter	Value					Description
	Default	OPF	NCL1A	BGN	Synthetic	
*r*_*s*_	6	0	0	0, 4, 8, 12	0, 4	Erosion radius [voxels]
*σ*	{2, 4, 6}	{2},{2, 4},{2, 4, 6}	{2},{2, 4},{2, 4, 6}	{2},{2, 4},{2, 4, 6}	{2, 4, 6}	Scale combinations [voxels]
*τ*	10	4, 6, 8, 10, 12	6, 8, 10	6, 8, 10	6, 8, 10	Local maxima tolerance [8-bit scale]
*N*	20	20	20	20	20	Number of samples
*κ*	3	3	3	3	3	Circular variance [voxels]
*d*	3	3	3	3	3	Tracing step size [voxels]
*ζ*	1	1	1	1	1	Scale variance [voxels]
*K*	20	20, 50	20, 50	20	20, 50	Likelihood sensitivity
$c_{\min }$	0.5	0.4, 0.5	0.3, 0.4, 0.5	0.3, 0.4, 0.5	0.3, 0.4, 0.5	Correlation threshold
*L*	200	200	200	200	200	Iteration limit
*δ*_*n*_	*δ*_9_ = 4	*δ*_9_ = 4	*δ*_9_ = 4	*δ*_1_ = 3, *δ*_9_ = 4	*δ*_1_ = 3, *δ*_9_ = 4	Node density limit [count/volume]
*r*_*g*_	2	2	2	2	2	Grouping radius [voxels]

The ordering is according to first mention in the main text

### Tree Construction

The final step of our method is the construction of a graph representing the complete neuronal arbor. This is facilitated by the bidirectional connectivity of the group nodes in $\hat {\mathcal {N}}$. However, similar to a real neuron, the final graph must be a tree, in which the nodes are unidirectionally linked (Figs. 1f and 4d), as also required by the SWC file format for storing digital neuron reconstructions (Stockley et al. 1993; Cannon et al. 1998). Starting from the soma node, or from the group node with the highest cross-correlation value if no soma was found in the image, the nodes in $\hat {\mathcal {N}}$ are iteratively traversed using a breadth-first search (BFS) algorithm. In this process it is possible to discard any isolated branches and single-node terminal branches (false positives).

### Implementation Details

Our method, which we call Probabilistic Neuron Reconstructor (PNR), was implemented in C++ as a plugin for the freely available and extendable bioimage visualization and analysis tool Vaa3D (Peng et al. 2010; 2014a).^2^ The source code of PNR is freely available for non-commercial use.^3^ As mentioned in the preceding sections, the method has a number of free parameters, which are summarized in Table 1, where we also list default values.

## Experimental Results

The performance of our PNR method was evaluated using both synthetic and real fluorescence microscopy image stacks of single neurons and was compared to several alternative 3D neuron reconstruction methods that yielded favorable performance in the BigNeuron project (Peng et al. 2015a). These included the second all-path pruning method (APP2) (Xiao and Peng 2013), NeuroGPS-Tree (GPS) (Quan et al. 2016), BigNeuron’s minimum spanning tree (MST) method, and we also added our recently published alternative probabilistic method based on probability hypothesis density filtering (PHD) (Radojević and Meijering 2017a).

To quantify performance we adopted the commonly used measures of distance and overlap of neuron reconstructions with respect to the ground truth (in the case of synthetic images) or the gold-standard reconstructions obtained by manual annotation (in the case of real images). The distance measures were the average minimal reciprocal spatial distance (SD) between nodes in the reconstructions being compared, the substantial spatial distance (SSD) using only the nodes with a spatial distance larger than a threshold S, and the percentage of these substantially distant nodes (%SSD), all computed after densely resampling each reconstruction to reduce the distance between its adjacent nodes to one voxel (see Peng et al. 2010 for details). The overlap measures were precision (P), recall (R), and the F score (Powers 2011), computed from the numbers of true-positive (TP), false-positive (FP) and false-negative (FN) nodes according to the spatial distance threshold S.

All experiments were performed on a MacBook Pro with 2.2 GHz Intel Core i7 processor and 16 GB RAM memory to test the practicality of the methods on a typical computer system. For each method we optimized the score for each performance measure by exploring a grid of possible parameter values around the default ones (see Table 1 for our method and the cited papers for the other methods). To keep the experiments feasible, we set the maximum allowed processing time per stack and method to 2 hours. In the sequel, to save space, we show only the F scores (higher is better) and SSD scores (lower is better), while the P, R, SD, and %SSD scores are given in the supplement. Our conclusions are based on the complete body of results.

### Experiments on Synthetic Neuron Images

Prior to evaluating how well our method emulates expert manual reconstruction in real neuron images, we first performed a controlled experiment using synthetic neuron images, with known ground-truth reconstructions and predefined levels of signal-to-noise ratio (SNR) and inter-voxel correlation (COR). This allowed us to study the robustness of our method compared to the others as a function of these image quality factors. For this experiment we selected 10 neurons from the BigNeuron training data set (Peng et al. 2015a), representative of the range of morphological complexities in the data set, and for which node radius information (non-default) was available in the corresponding gold-standard reconstructions in SWC format.

**Fig. 5:**
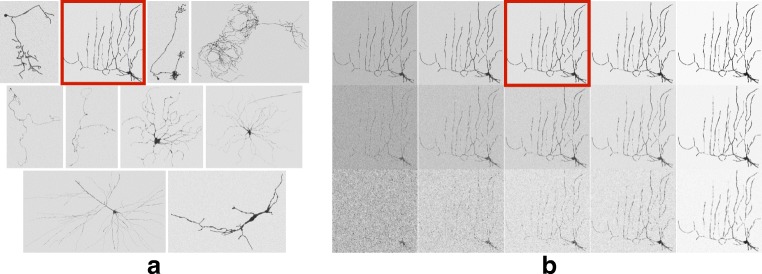
Illustration of the synthetic neuron data set used in the presented experiments. **a** Example images of the 10 selected neurons simulated at SNR = 4 and COR = 0.0. **b** Different simulations of the neuron indicated by the red outline in **(a)** for SNR = 2, 3, 4, 5, and 10 (from left to right) and COR = 0.0, 1.0, and 2.0 (from top to bottom). The marked image in **(b)** is the same as the marked image in **(a)**. All examples shown here are maximum intensity projections of the 3D synthetic images with inverted intensities for better visualization

We developed a plugin for ImageJ (Schneider et al. 2012) called SWC2IMG,^4^ which takes any SWC file as input and simulates fluorescence microscopy imaging of all neuronal branches in the file at a specified SNR and COR level, producing an image stack whose true digital reconstruction is the very input. It assumes that in practice, because of the relatively large spatial extent of even a single neuron with its complete arbor, the combination of optical magnification factor and digital image matrix size in real neuron images is typically such that the voxel size is larger than the point spread function (PSF), implying that the partial-volume effect of digitization is more prominent than the optical blurring by the microscope. Based on this, the plugin simulates the imaging simply by estimating for each voxel which fraction of its volume is occupied by the neuron. Next, it simulates noise by using the Poisson noise model representative of optical imaging, which defines SNR as the image intensity inside the neuron above the background, divided by the standard deviation of the noise inside (Sheppard et al. 2006). And finally, to allow for correlated signal and noise, which we found to improve the visual realism of the simulated images, the plugin also offers the possibility to apply Gaussian smoothing at a specified scale, being the COR parameter, while preserving the SNR level. Generally, the lower the SNR and/or the higher the COR level, the more challenging the data and the reconstruction problem.

Using this plugin we created a synthetic data set containing image stacks for a range of SNR and COR values for each neuron (Fig. 5). Specifically, we considered SNR = 1, 2, 3, 4, 5, 10, 20, and COR = 0, 0.5, 1, 1.5, 2. Thus, our synthetic data set consisted of 10 (neurons) × 7 (SNR levels) × 5 (COR levels) = 350 image stacks,^5^ which we attempted to reconstruct optimally using the five considered methods (APP2, GPS, MST, PHD, PNR) and a parameter grid-search approach. However, some of the images were very challenging, especially the ones with many branches and low SNR or high COR values, causing the methods to sometimes require excessive computation times or even to get stuck altogether. Because of the mentioned time constraint, not all methods were able to complete all the reconstructions, and it turned out that only 7 out of the 10 neurons could be reconstructed by all the methods for all SNR and COR values. Therefore we present the results only for those.

**Fig. 6:**
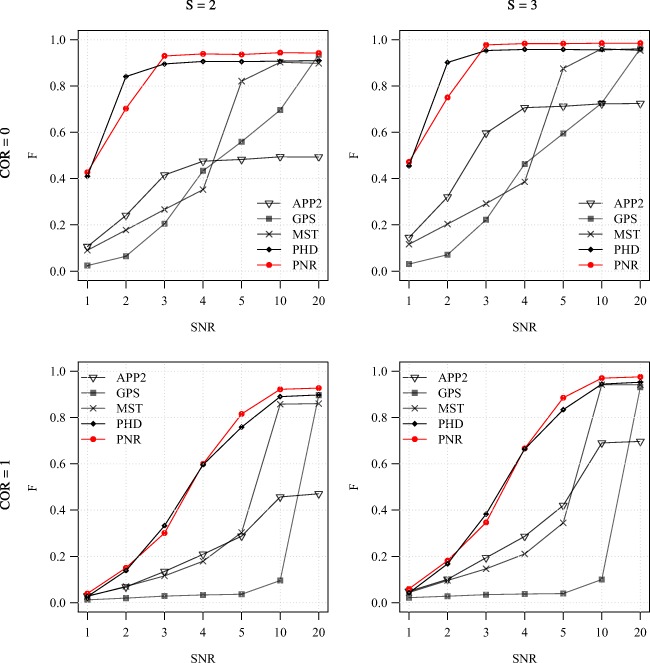
Average F score of the methods for the synthetic images as a function of SNR. Examples are shown for COR = 0 (top) and 1 (bottom) in combination with S = 2 (left) and 3 (right)

**Fig. 7:**
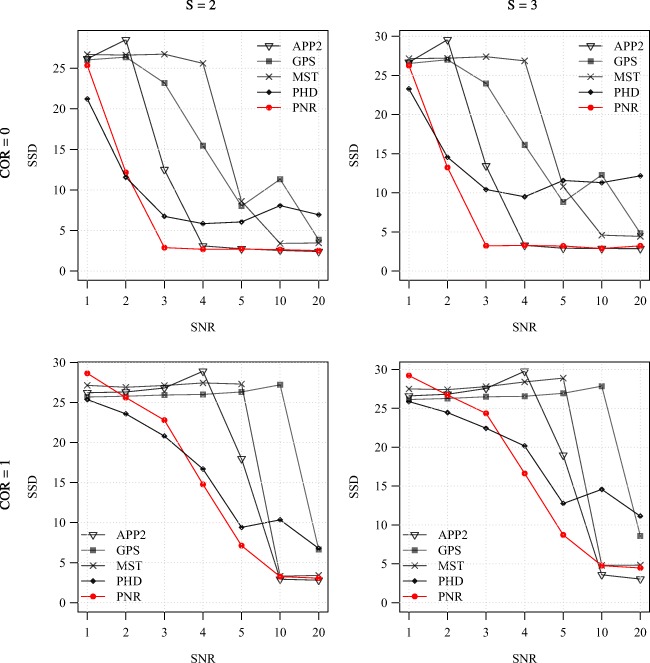
Average SSD score of the methods for the synthetic images as a function of SNR. Examples are shown for COR = 0 (top) and 1 (bottom) in combination with S = 2 (left) and 3 (right)

**Fig. 8:**
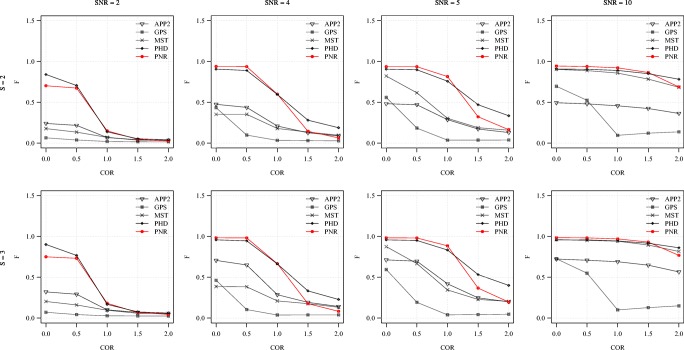
Average F score of the methods for the synthetic images as a function of COR. Examples are shown for S = 2 (top) and 3 (bottom) in combination with SNR = 2, 4, 5, 10 (left to right)

**Fig. 9:**
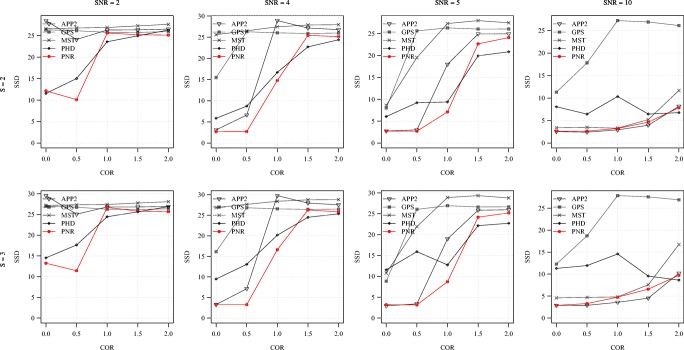
Average SSD score of the methods for the synthetic images as a function of COR. Examples are shown for S = 2 (top) and 3 (bottom) in combination with SNR = 2, 4, 5, 10 (left to right)

**Fig. 10:**
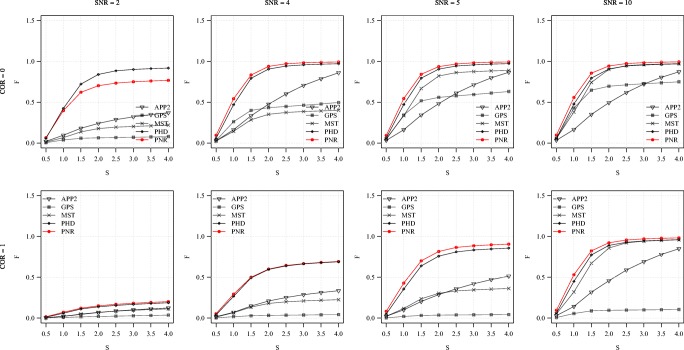
Average F score of the methods for the synthetic images as a function of S. Examples are shown for COR = 0 (top) and 1 (bottom) in combination with SNR = 2, 4, 5, 10 (left to right)

**Fig. 11:**
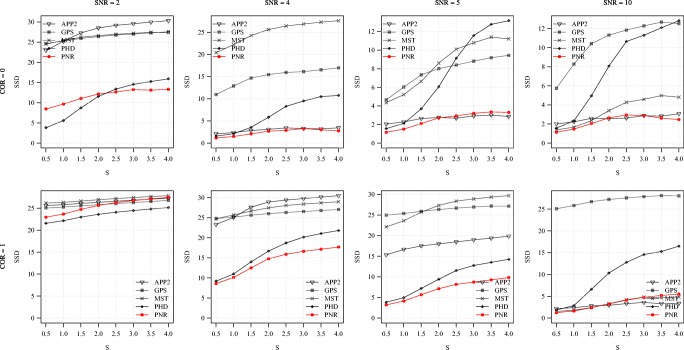
Average SSD score of the methods for the synthetic images as a function of S. Examples are shown for COR = 0 (top) and 1 (bottom) in combination with SNR = 2, 4, 5, 10 (left to right)

From the average F and SSD scores of the methods as a function of SNR for a few sample values of COR and S (Figs. 6 and 7) we generally observe that, as expected, increasing the SNR improves the performance of all methods (increasing F and decreasing SSD scores). We also note that the two probabilistic methods (PHD and PNR) are more robust against noise (especially according to F) and that our proposed method (PNR) is often superior overall. The results also show that, as expected, increasing the value of COR (which yields more difficult images) has a strong negative impact on the performance of all methods (lower F and higher SSD scores for the same SNR). This is confirmed when looking more in-depth at the results as a function of COR (Figs. 8 and 9). Additionally, again as expected, in all cases we observe that increasing the value of S (meaning being more lenient in matching reconstructions to the ground truth) may also strongly affect the scores of all methods (meaning higher F scores, but in this case also higher SSD scores, as the latter includes only node distances larger than S). This is confirmed when looking explicitly at the performance of the methods as a function of S (Figs. 10 and 11). These results reveal that both the absolute and the relative performance of different methods being compared may depend on S. This is an important observation, since in all studies we are aware of, the somewhat arbitrary value of S = 2 is taken for granted in calculating performance and ranking the methods. Our results (Figs. 10 and 11) show that taking other values of S may yield a different ranking. Notwithstanding this finding, our results also show that under most experimental conditions (SNR, COR, S), the proposed method (PNR) yields superior results. While our previous probabilistic neuron tracing method (PHD) (Radojević and Meijering 2017a) is often a strong competitor, the results indicate that our new method is more favorable, which we believe can be ascribed to its better model for seed point extraction and branch tracing.

**Fig. 12:**
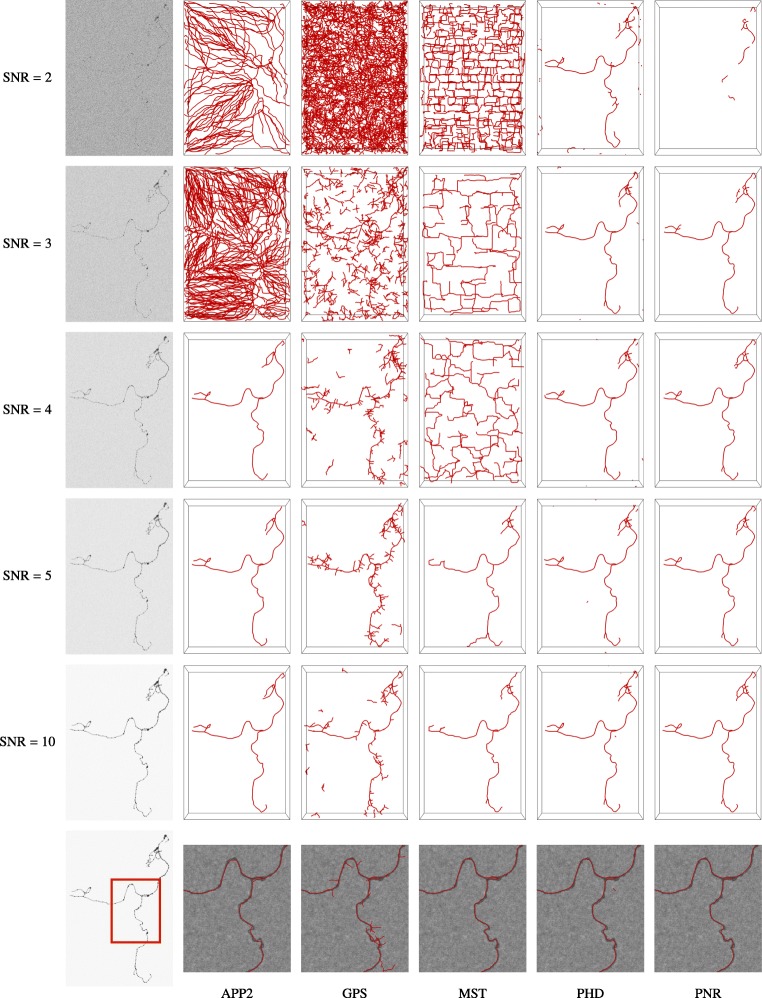
Visual comparison of neuron reconstructions produced by the considered methods from synthetic image stacks of a single neuron at different SNR levels. The image stacks (generated used COR = 0) are shown as inverted maximum intensity projections (left column) and the reconstructions of the different methods (remaining columns) are shown in red as surface renderings

Together, the results of our experiments on synthetic neuron images suggest that tracing the image structures repeatedly and in a statistically independently way, indeed yields more evidence about the underlying neuron branches and leads to better reconstructions. This also follows from a visual comparison of the reconstructions (Fig. 12). Especially at low SNRs, pruning and fast-marching based methods tend to oversegment the images, while our probabilistic methods still perform relatively well regardless. Even at high SNRs, when most of the methods perform comparably, our proposed method follows the branch structures more closely (see zooms in the last row of Fig. 12).

**Fig. 13:**
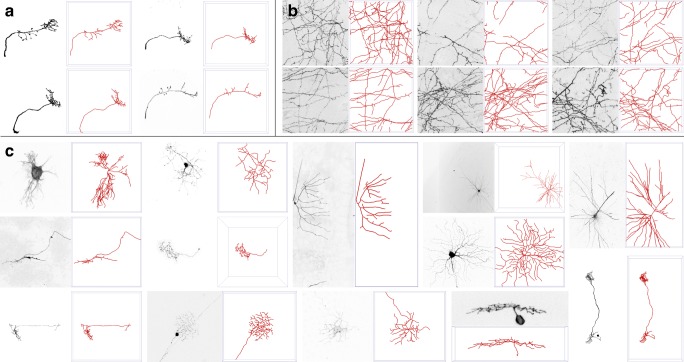
Illustration of the real neuron image data sets used in the presented experiments. Examples are shown of **a** the OPF data set (4 of 9 stacks), **b** the NCL1A data set (6 of 16 stacks), and **c** the BGN data set (13 of 76 stacks). Each example shows the maximum intensity projection of the image stack (left panel) but with inverted intensities for better visualization, and the corresponding manual reconstruction (right panel) as a surface rendering (in red), both generated using Vaa3D (Peng et al. 2010)

**Fig. 14:**
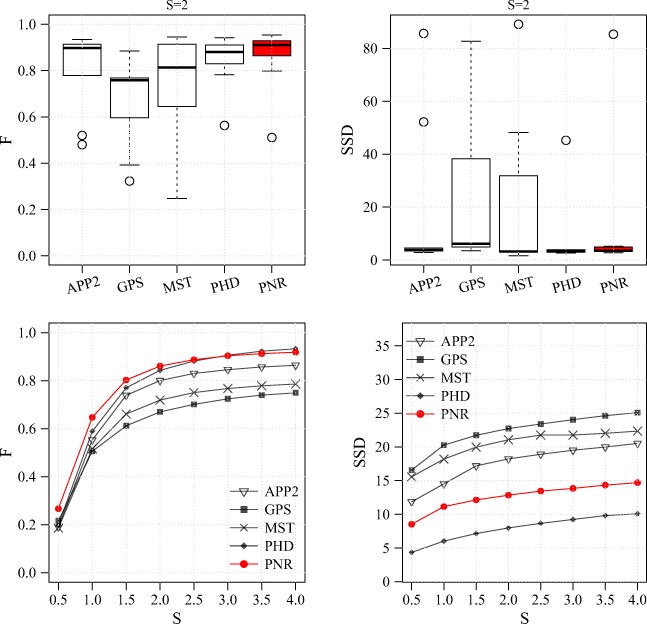
Performance comparison for the OPF data set. Results are shown for the F measure (left column) and SSD measure (right column) and in the form of distributions for S = 2 (standard R box plots in top row) and averages as a function of S (bottom row)

**Fig. 15:**
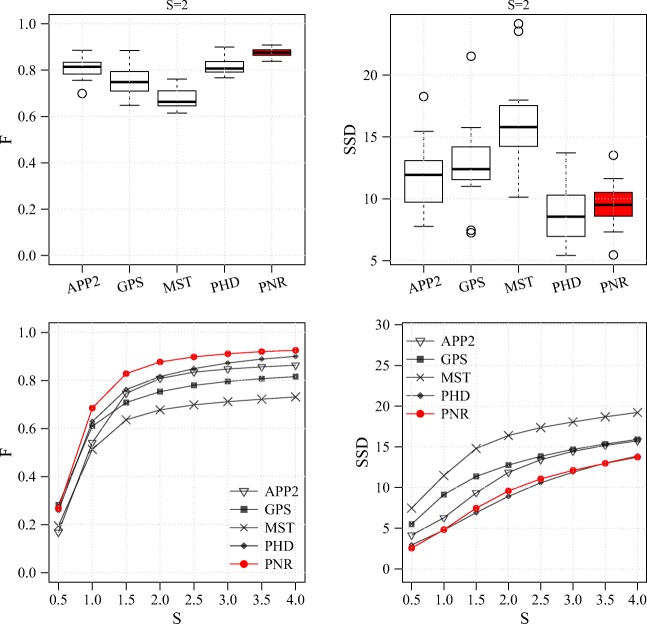
Performance comparison for the NCL1A data set. Results are shown for the F measure (left column) and SSD measure (right column) and in the form of distributions for S = 2 (standard R box plots in top row) and averages as a function of S (bottom row)

**Fig. 16:**
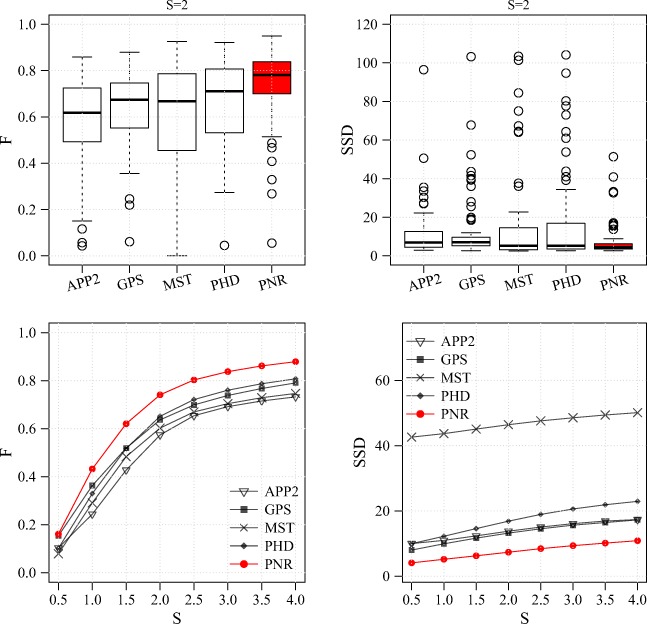
Performance comparison for the BGN data set. Results are shown for the F measure (left column) and SSD measure (right column) and in the form of distributions for S = 2 (standard R box plots in top row) and averages as a function of S (bottom row)

**Fig. 17:**
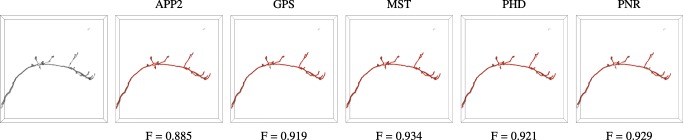
Example neuron reconstructions of an image stack from the OPF data set. Shown are the original arbor (volume rendering on the left) and the reconstructions (overlaid surface renderings in red) of the different methods (indicated at the top) corresponding to the best F score (given below each reconstruction) for S = 2 with respect to the available manual reconstruction

**Fig. 18:**
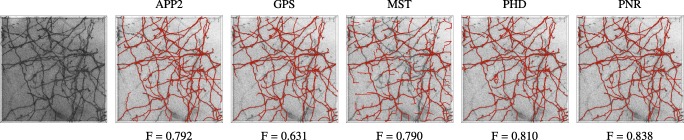
Example neuron reconstructions of an image stack from the NCL1A data set. Shown are the original arbor (volume rendering on the left) and the reconstructions (overlaid surface renderings in red) of the different methods (indicated at the top) corresponding to the best F score (given below each reconstruction) for S = 2 with respect to the available manual reconstruction

### Experiments on Real Neuron Images

In addition to synthetic data we also used three real neuron image data sets to evaluate the absolute and relative performance of our method. The first two are the olfactory projection fibers (OPF) data set (9 image stacks) and neocortical layer-1 axons (NCL1A) data set (16 image stacks) from the DIADEM challenge (Brown et al. 2011), and the third is part of the BigNeuron (BGN) training data set (76 image stacks) (Peng et al. 2015a), all imaged with fluorescence microscopy (confocal or two-photon) and manually annotated as described in detail in the cited works and corresponding resources. Being the smallest of the three, in terms of both neuronal volume and complexity, OPF is probably the most often used data set in the field. NCL1A is often used as it contains neuronal network-like structures and no clear somas. And BGN is the largest, most diverse, and thus most challenging data set for evaluating neuron reconstruction methods. Together, the 100+ image stacks in these data sets have a wide variety of image qualities and volumes (10 MB to 2 GB per stack) and portray a wide range of neuronal shapes and complexities (Fig. 13), representative of many studies. For some stacks in the BGN data set, the voxel size was unknown, and in these cases we used a default x:y:z voxel aspect ratio of 1:1:2, reflecting the typically lower resolution in the depth dimension. Also, because of the mentioned processing time constraint, 3 of the 76 image stacks could not be reconstructed by all methods (see Supplementary Fig. ?? for these and other hard cases), so the presented results are based on the remaining 73.

**Fig. 19:**
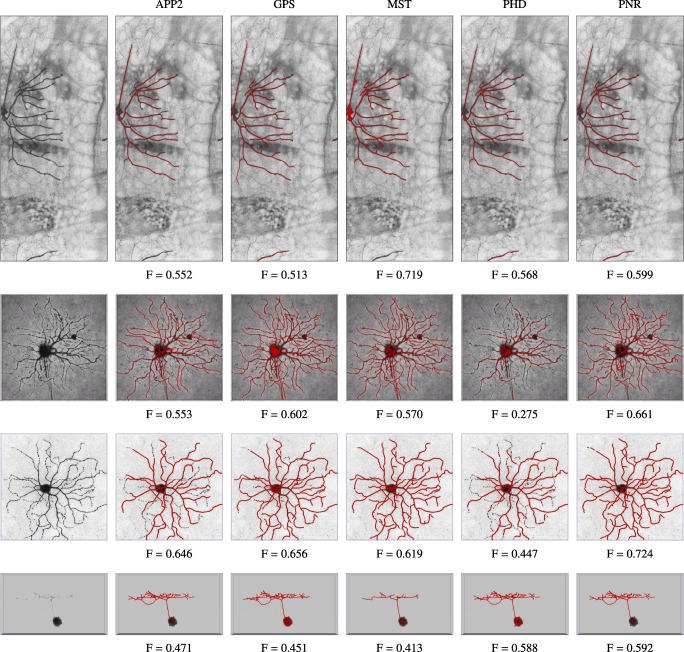
Example neuron reconstructions of four image stacks from the BGN data set. Shown are the original arbors (volume renderings on the left) and the reconstructions (overlaid surface renderings in red) of the different methods (indicated at the top) corresponding to the best F score (given below each reconstruction) for S = 2 with respect to the available manual reconstruction

From the results of the experiments on these three real data sets (Figs. 14, 15 and 16) we observe that, as in the experiments on synthetic data, the probabilistic methods PHD and PNR typically show superior performance in terms of both F and SSD score. Of these two methods, our proposed PNR method consistently shows the smallest performance spread, indicating it is more robust than our previously published PHD method. For the BGN data set, being the most diverse of the three, the performance spread (including outliers) of all methods is the largest, and the increase in performance as a function of S is the smallest, as expected. Nevertheless, the PNR method consistently shows the best overall performance especially for this data set. In other words, for any given data set similar to those considered in this study, PNR is the favorable method a priori. Obviously this does not necessarily mean that PNR will give the best reconstruction for each and every image stack in the data set, but simply that the chances are higher. This is confirmed when we look at a few example image stacks from the three data sets and the corresponding best reconstructions produced by the different methods by maximizing the F score in the parameter grid search (Figs. 17, 18 and 19). As these examples show, although PNR often outperforms the other methods, in specific cases one of the other methods may give better results.

Finally we investigated the sensitivity of PNR with respect to two of its parameters that one might suspect to be rather critical. The first is the noise tolerance parameter *τ* used to prune insignificant local maxima in the seed extraction (Seed Extraction). To obtain the best possible results while keeping the computation times manageable, we considered different sets of values for this parameter, depending on the data set (Table 1). For example, in the case of the relatively small-sized OPF data set we considered values *τ* = 4,6,8,10,12, while for the larger NCL1A and BGN data sets and the synthetic images we examined *τ* = 6,8,10. The results (Supplementary Figs. ??-??) show that *τ* is in fact not a very sensitive parameter of the method and that the suggested default value is a suitable choice. The second parameter is the node density limit *δ*_*n*_ used to terminate the tracing (Branch Tracing). Here, too, we considered different sets of values depending on the data set, for *n* = 5 and *n* = 9. The results (Supplementary Fig. ??) show that the method is also not very sensitive to this parameter and its default value is suitable. Notice that due to the probabilistic nature of the method there is inherently some randomness in the results. But altogether we believe the results justify the conclusion that PNR is a robust method and a valuable addition to the neuron reconstruction toolbox.

## Conclusions

We have presented a new fully automated probabilistic neuron reconstruction method (PNR) based on sequential Monte Carlo filtering. It traces individual neuron branches from automatically detected seed points repeatedly but statistically independently to acquire more evidence and to be more robust to noise and other artifacts. The traces are subsequently refined, merged, and put into a tree representation for further analysis. We evaluated the method on both synthetic and real neuron images and compared it to various other state-of-the-art neuron reconstruction methods (APP2, GPS, MST, PHD) using commonly used quantitative performance measures (we presented F and SSD scores). To obtain realistic synthetic data we developed a novel simulator (SWC2IMG) that can turn any given SWC file into an image stack of specified quality whose ground truth reconstruction is the input. For the evaluation on real data we used about 100 single-neuron fluorescence microscopy image stacks of widely varying quality and complexity, with corresponding manual reconstructions serving as the gold standard, from three different data sets used in the DIADEM and BigNeuron studies. The results show conclusively that the proposed method is generally favorable and also outperforms our own alternative neuron reconstruction method based on probability hypothesis density (PHD) filtering we presented recently. Nevertheless, there still remains much room for further improvement, as none of the quantitative scores were near perfect for any of the considered methods even for high SNR levels and very lenient distance thresholds. Possible directions for future work within the presented probabilistic framework would be to explore other state transition and measurement models. Alternatively, since no single method always performs best on all images of a given data set, and the results of different methods are likely complementary, another possible direction could be to combine multiple methods either during tracing or in a post-processing step. The latter approach is already being explored in the BigNeuron project. But regardless of the outcome of this effort we conclude that the method proposed in this paper may already prove to be of great use in many cases. Our software implementation of the method will be made freely available for non-commercial use upon publication.

## Information Sharing Statement

The source code of the presented method is freely available for non-commercial use from https://bitbucket.org/miroslavradojevic/pnr.

## Electronic supplementary material

Below is the link to the electronic supplementary material.

(PDF 5.58 MB)

## Footnotes

1
http://bitbucket.org/miroslavradojevic/vess

2
http://vaa3d.org

3
https://bitbucket.org/miroslavradojevic/pnr

4
https://github.com/imagescience/SWC2IMG

5
https://bitbucket.org/miroslavradojevic/pnr
