# Supplementary material for: Automated Neuron Reconstruction from 3D Fluorescence Microscopy Images Using Sequential Monte Carlo Estimation
Source: Neuroinformatics. 2018 Dec 12;17(3):423–42. doi: 10.1007/s12021-018-9407-8 (PMC6594993; doi:10.1007/s12021-018-9407-8)
Supplement: Supplementary file 1 — (PDF 5.58 MB) [file 12021_2018_9407_MOESM1_ESM.pdf]

Automated Neuron Reconstruction from 3D Fluorescence Microscopy Images using Sequential Monte Carlo Estimation

Miroslav Radojević and Erik Meijering

Supplementary Information

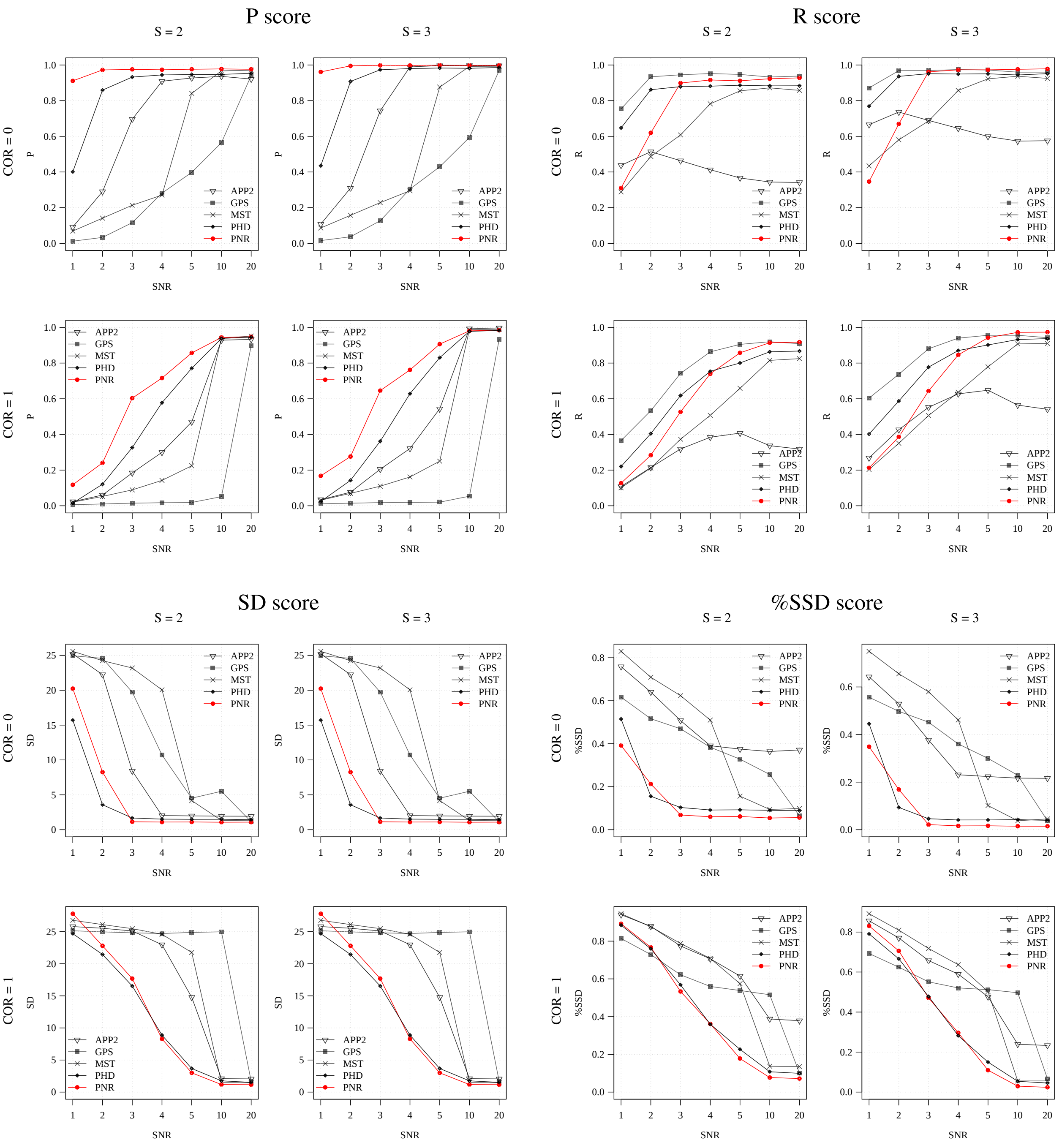

Figure S1: Average P, R, SD, and %SSD score of the methods for the synthetic images as a function of SNR. For each of the four measures examples are shown for COR = 0 (top) and 1 (bottom) in combination with S = 2 (left) and 3 (right).

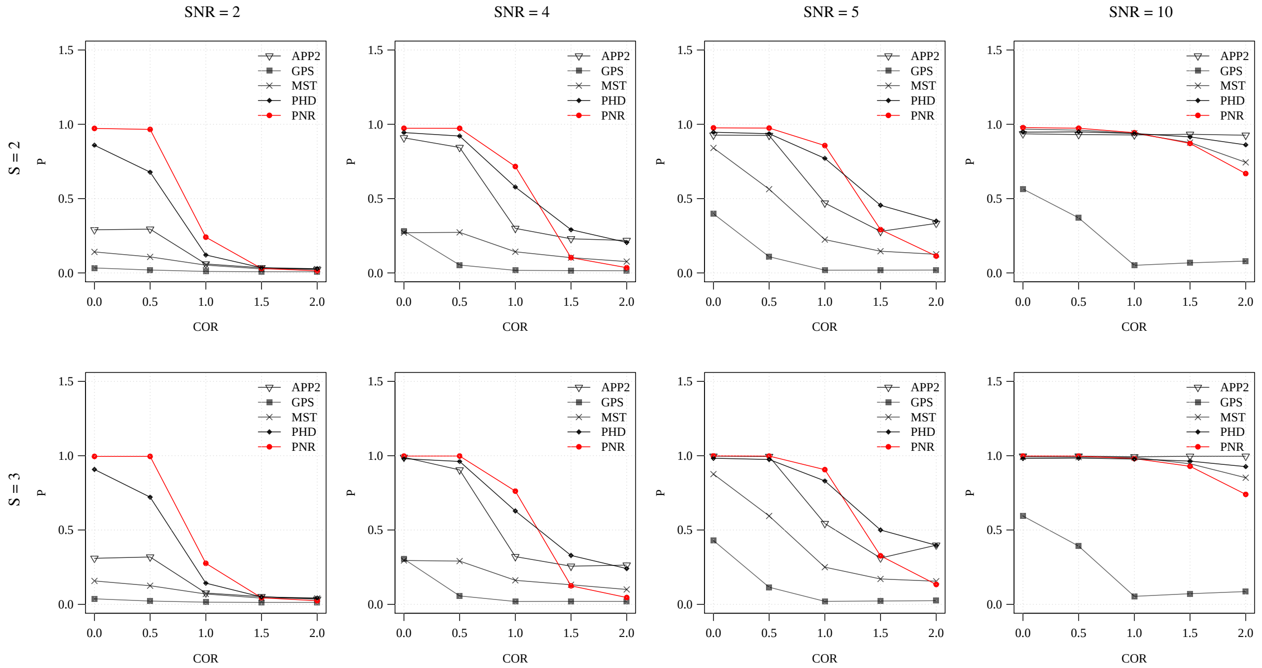

Figure S2: Average P score of the methods for the synthetic images as a function of COR. Examples are shown for S = 2 (top) and 3 (bottom) in combination with SNR = 2, 4, 5, 10 (left to right).

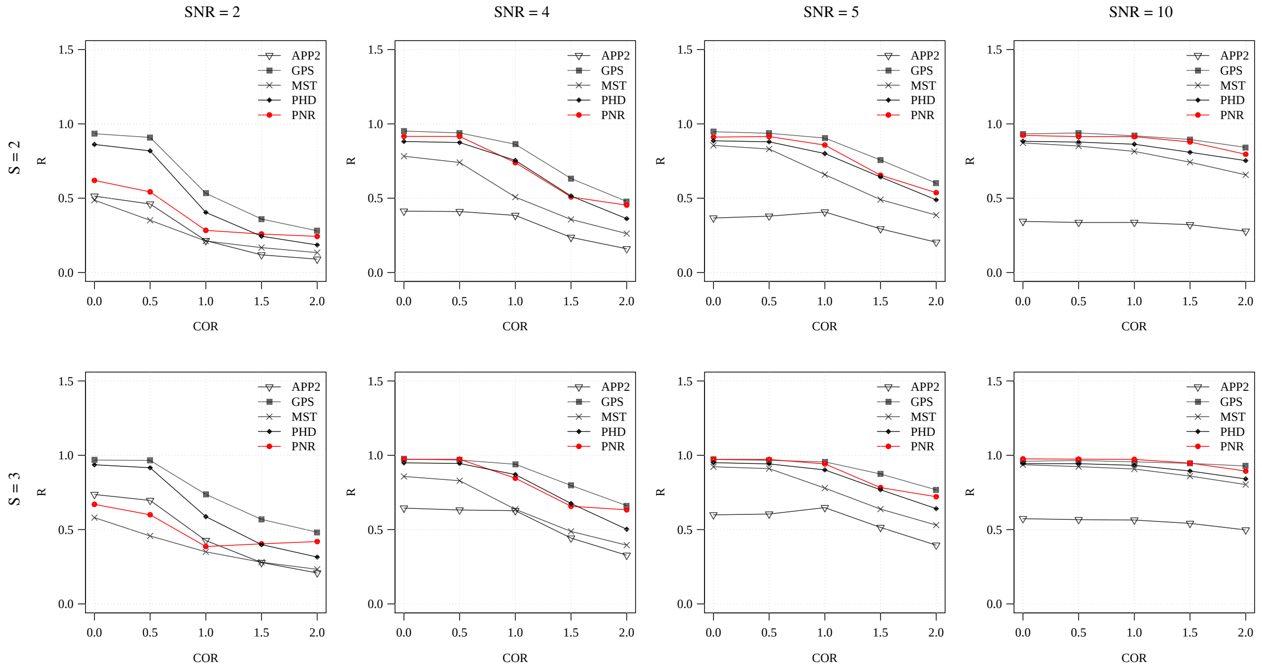

Figure S3: Average R score of the methods for the synthetic images as a function of COR. Examples are shown for S = 2 (top) and 3 (bottom) in combination with SNR = 2, 4, 5, 10 (left to right).

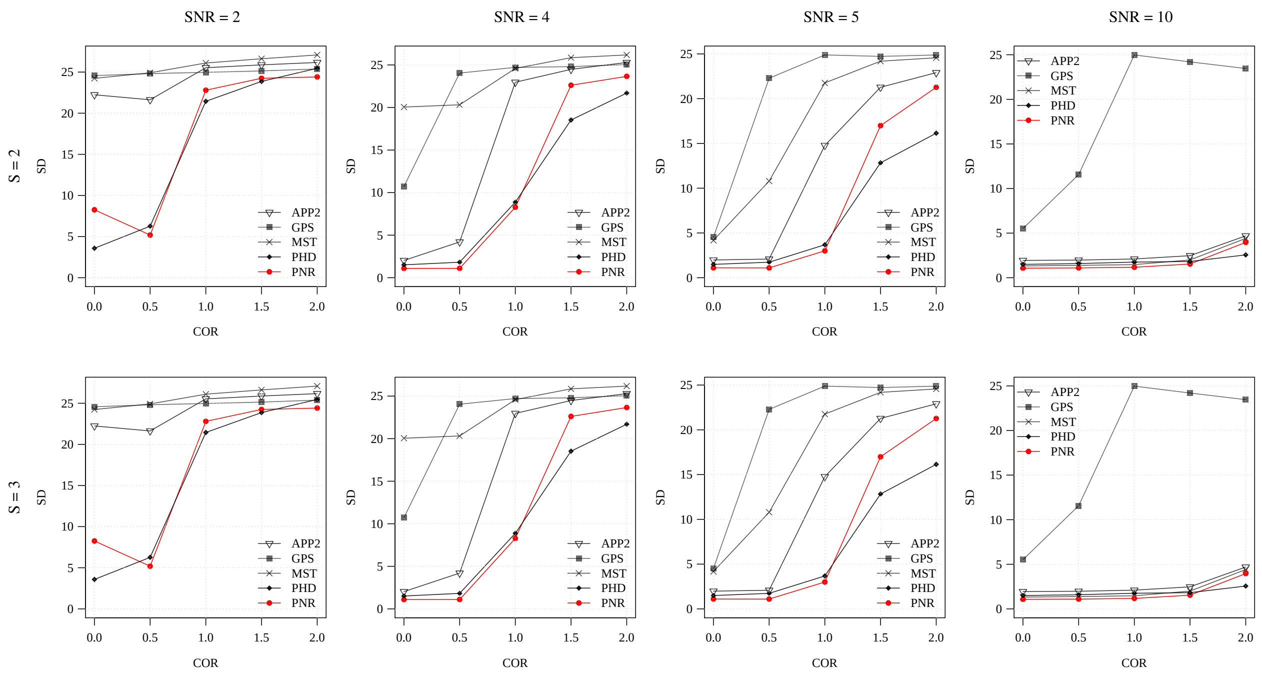

Figure S4: Average SD score of the methods for the synthetic images as a function of COR. Examples are shown for S = 2 (top) and 3 (bottom) in combination with SNR = 2, 4, 5, 10 (left to right).

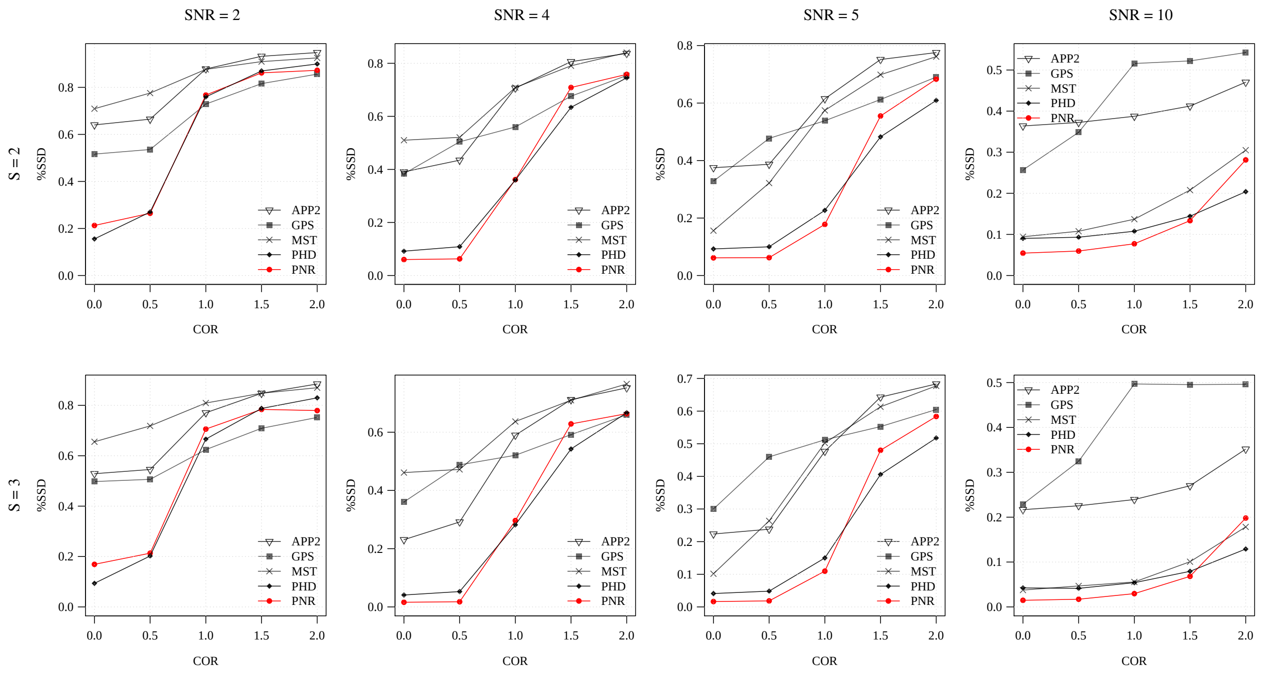

Figure S5: Average %SSD score of the methods for the synthetic images as a function of COR. Examples are shown for S = 2 (top) and 3 (bottom) in combination with SNR = 2, 4, 5, 10 (left to right).

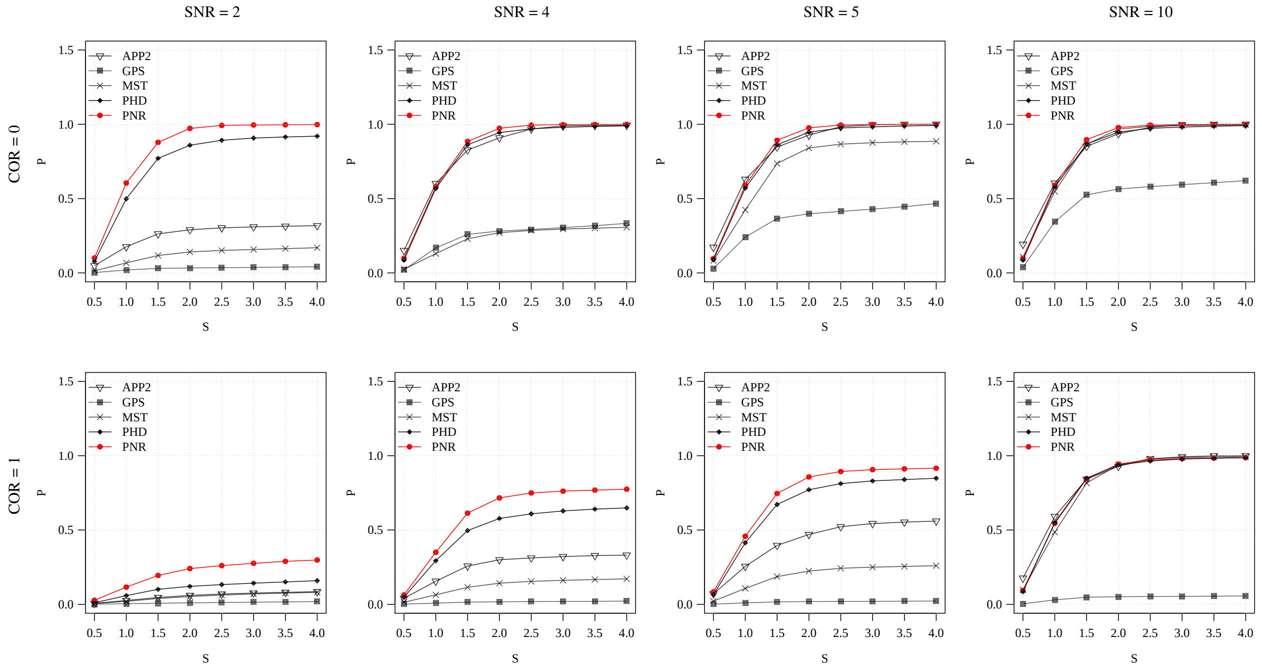

Figure S6: Average P score of the methods for the synthetic images as a function of S. Examples are shown for COR = 0 (top) and 1 (bottom) in combination with SNR = 2, 4, 5, 10 (left to right).

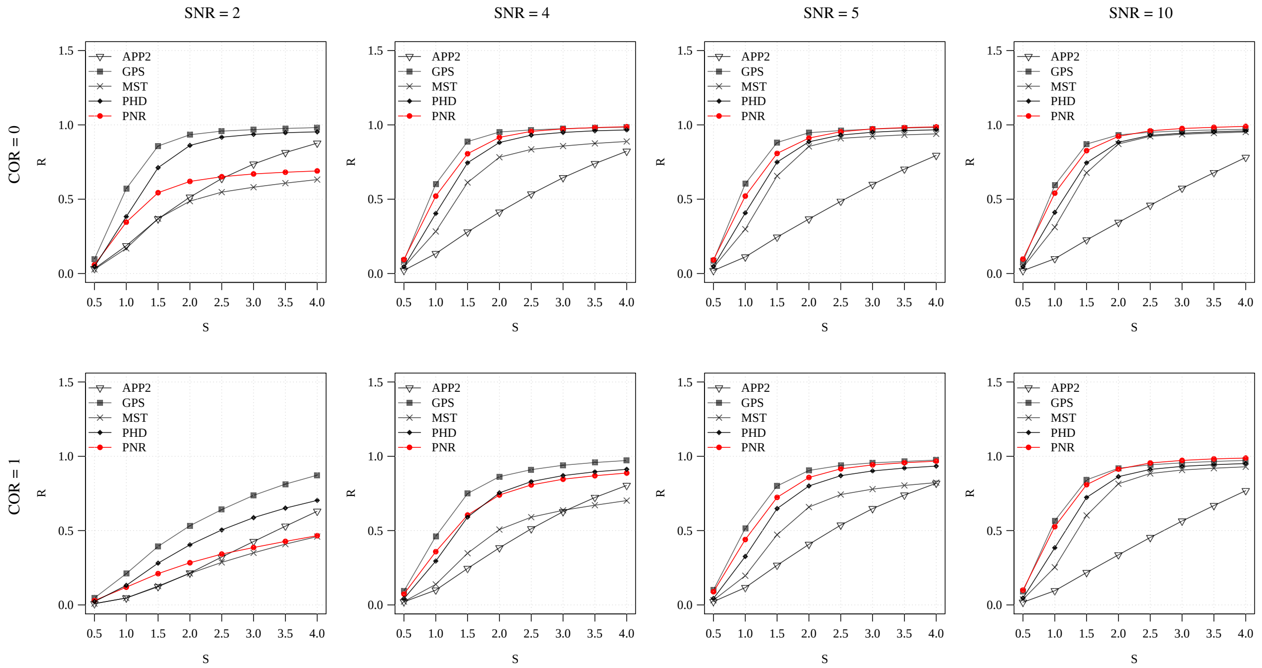

Figure S7: Average R score of the methods for the synthetic images as a function of S. Examples are shown for COR = 0 (top) and 1 (bottom) in combination with SNR = 2, 4, 5, 10 (left to right).

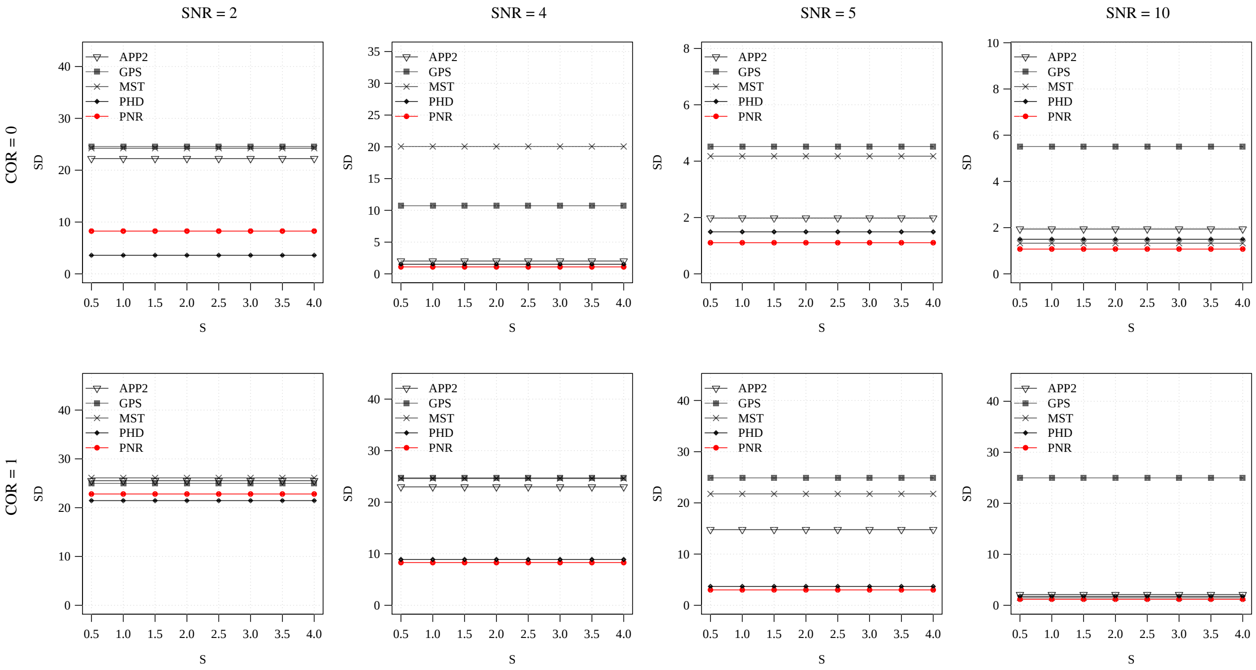

Figure S8: Average SD score of the methods for the synthetic images as a function of S. Examples are shown for COR = 0 (top) and 1 (bottom) in combination with SNR = 2, 4, 5, 10 (left to right). The plots reflect the fact that by definition SD is independent of S.

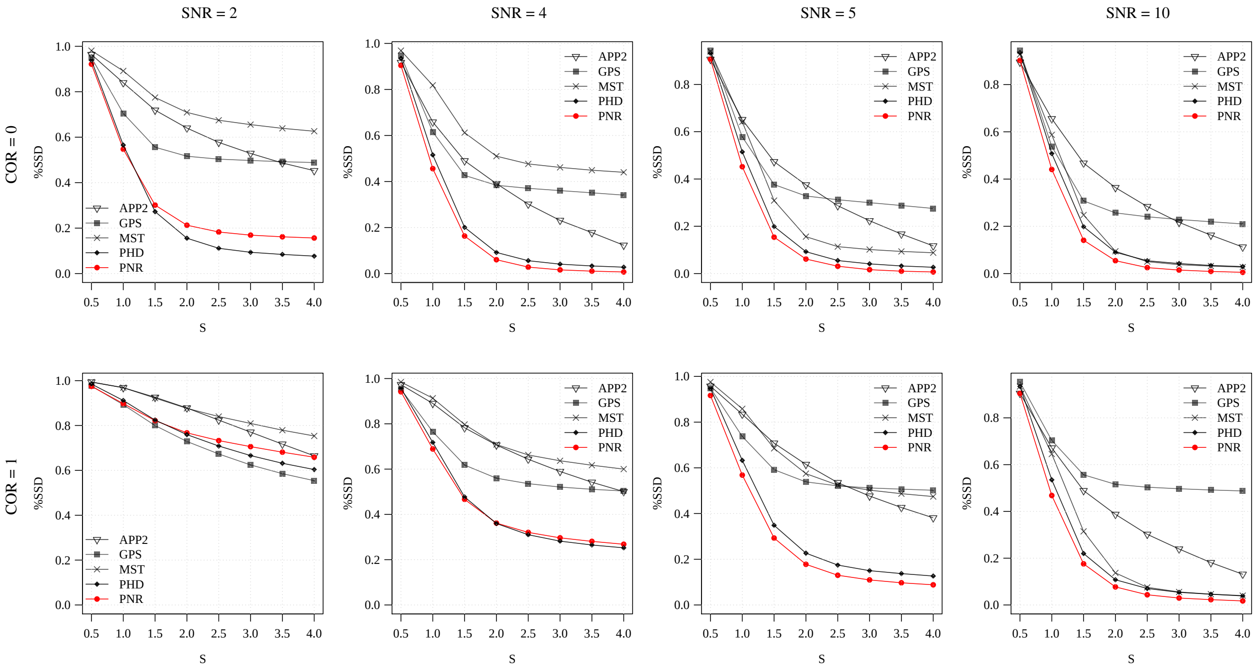

Figure S9: Average %SSD score of the methods for the synthetic images as a function of S. Examples are shown for COR = 0 (top) and 1 (bottom) in combination with SNR = 2, 4, 5, 10 (left to right).

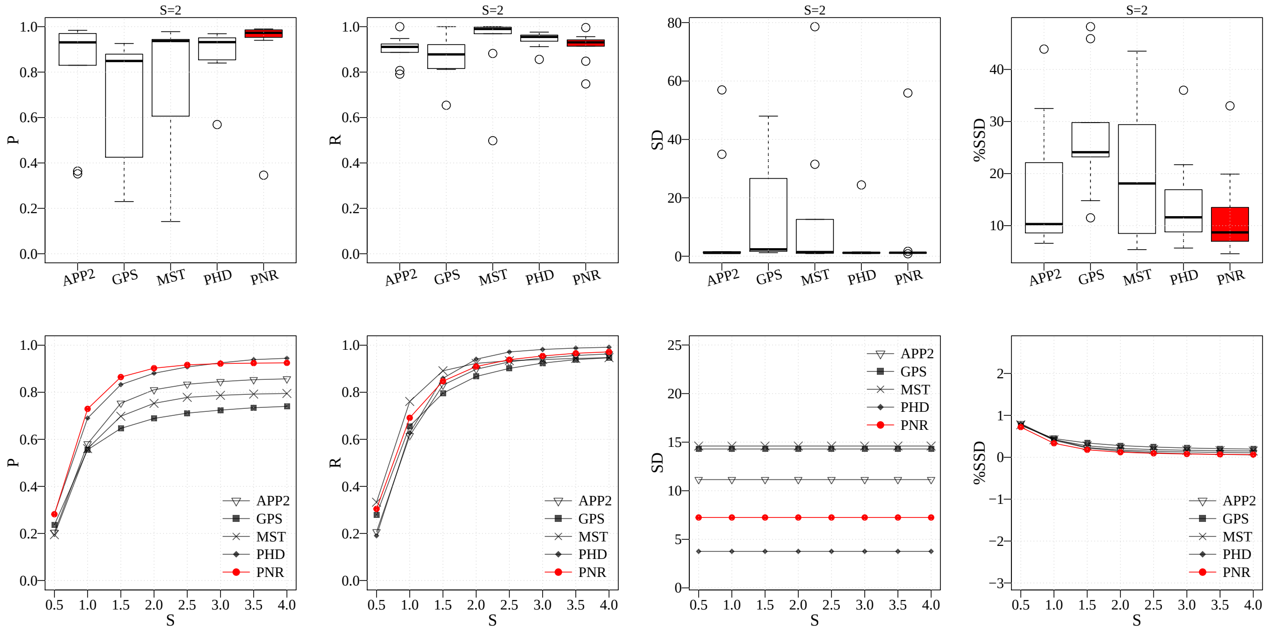

Figure S10: Performance comparison for the OPF data set. Results are shown for the precision (P), recall (R), spatial distance (SD), and significant spatial distance percentage (%SSD) (columns left to right). Measures are presented in the form of distributions for S = 2 (standard R box plots in top row) and averages as a function of S (bottom row).

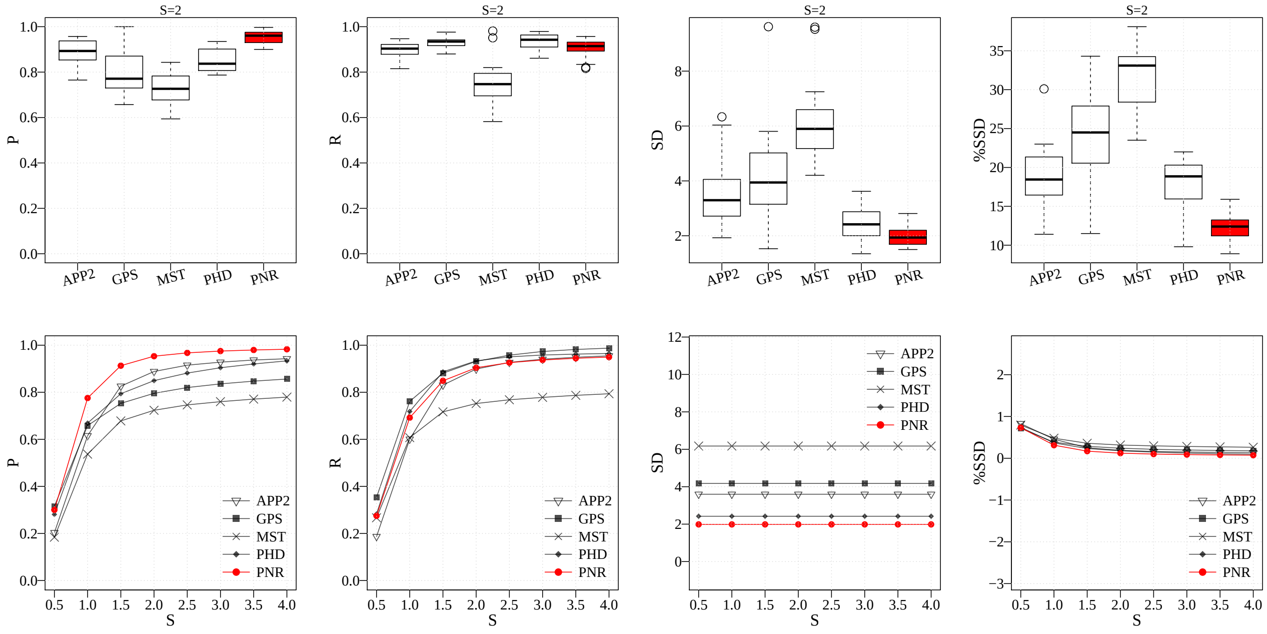

Figure S11: Performance comparison for the NCL1A data set. Results are shown for the precision (P), recall (R), spatial distance (SD), and significant spatial distance percentage (%SSD) (columns left to right). Measures are presented in the form of distributions for S = 2 (standard R box plots in top row) and averages as a function of S (bottom row).

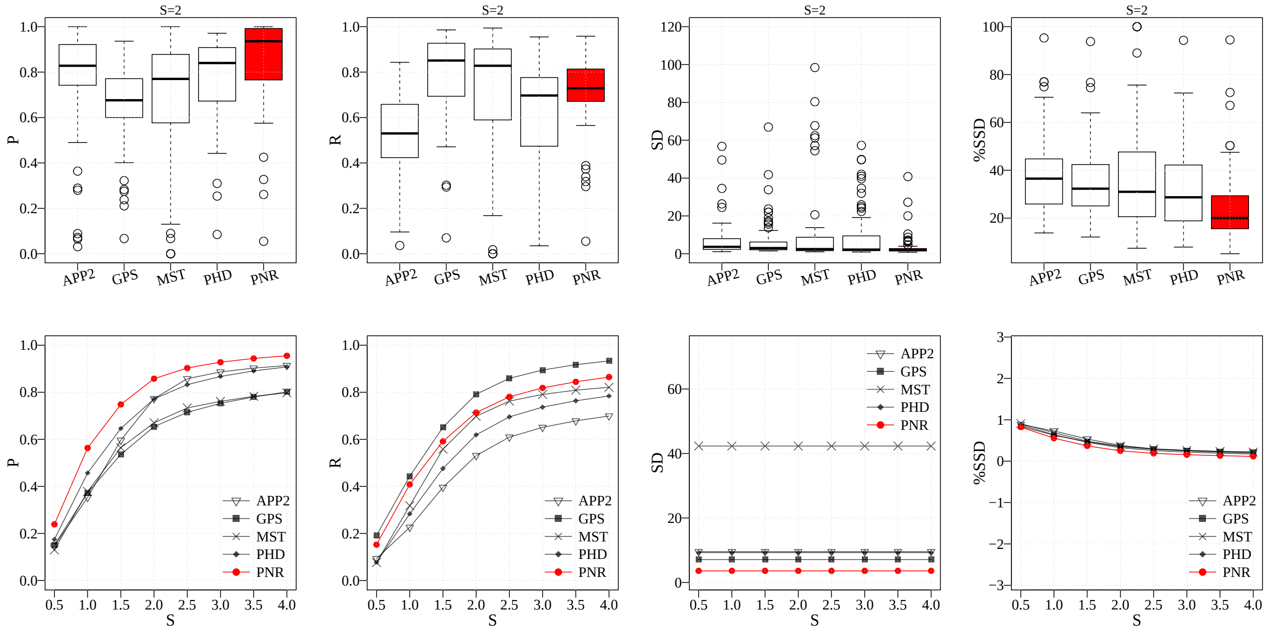

Figure S12: Performance comparison for the BGN data set. Results are shown (columns, left to right) for the precision (P), recall (R), spatial distance (SD), and significant spatial distance percentage (%SSD). Measures are presented in the form of distributions for S = 2 (standard R box plots in top row) and averages as a function of S (bottom row).

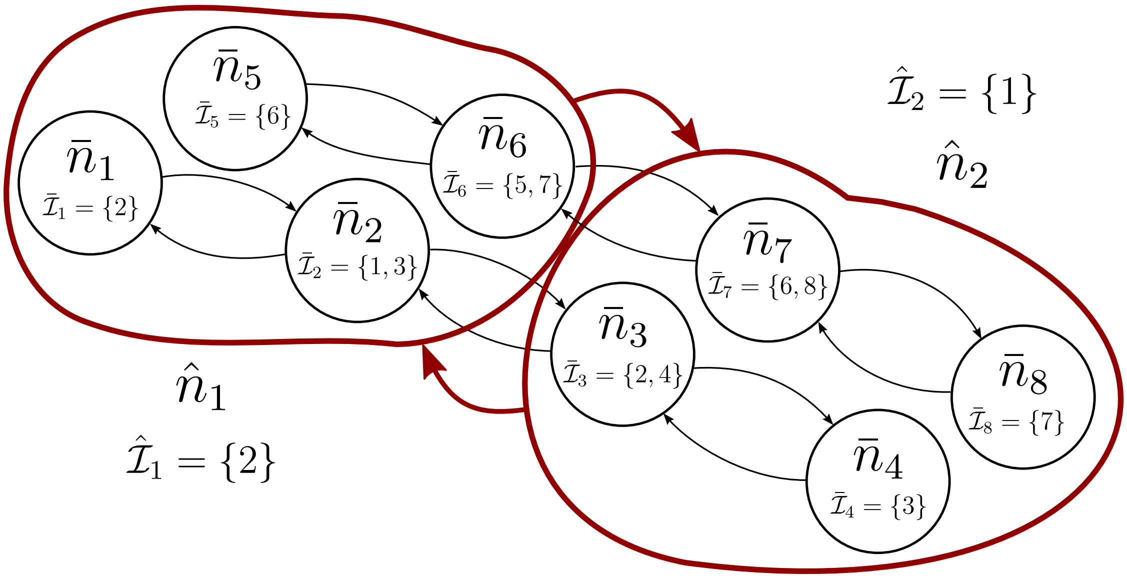

Figure S13: Example of node grouping. Here two traces, $\{\bar{n}_1, \bar{n}_2, \bar{n}_3, \bar{n}_4\}$ and $\{\bar{n}_5, \bar{n}_6, \bar{n}_7, \bar{n}_8\}$, are to become one single trace, $\{\hat{n}_1, \hat{n}_2\}$. Connections $\bar{\mathcal{I}}$ between the nodes $\bar{n}$ of the individual traces are mapped to connections $\hat{\mathcal{I}}$ between the group nodes $\hat{n}$ of the final single trace in Algorithm 1:

$\bar{\mathcal{N}} = \{\{\bar{n}_1^1,\ \bar{n}_2^1,\ \bar{n}_3^1,\ \bar{n}_4^1\},\ \{\bar{n}_1^2,\ \bar{n}_2^2,\ \bar{n}_3^2,\ \bar{n}_4^2\}\} = \{\bar{n}_1,\ \bar{n}_2,\ \bar{n}_3,\ \bar{n}_4,\ \bar{n}_5,\ \bar{n}_6,\ \bar{n}_7,\ \bar{n}_8\}$

$\bar{\mathcal{I}} = \{1:\{2\},\ 2:\{1,3\},\ 3:\{2,4\},\ 4:\{3\},\ 5:\{6\},\ 6:\{5,7\},\ 7:\{6,8\},\ 8:\{7\}\}$

$\Downarrow$ grouping

$\hat{\mathcal{I}}_1 = \{\{2\},\{1,3\},\{6\},\{5,7\}\}$

$\hat{\mathcal{I}}_2 = \{\{2,4\},\{3\},\{6,8\},\{7\}\}$

$\Downarrow$ node to group mapping

$G = \{1:1,\ 2:1,\ 3:2,\ 4:2,\ 5:1,\ 6:1,\ 7:2,\ 8:2\}$

$\Downarrow$ apply mapping

$\hat{\mathcal{I}}_1 = \{\{1\},\{1,2\},\{1\},\{1,2\}\}$

$\hat{\mathcal{I}}_2 = \{\{1,2\},\{2\},\{1,2\},\{2\}\}$

$\Downarrow$ remove duplicates

$\hat{\mathcal{I}}_1 = \{1,2\}$

$\hat{\mathcal{I}}_2 = \{1,2\}$

$\Downarrow$ remove self-links

$\hat{\mathcal{I}}_1 = \{2\}$

$\hat{\mathcal{I}}_2 = \{1\}$

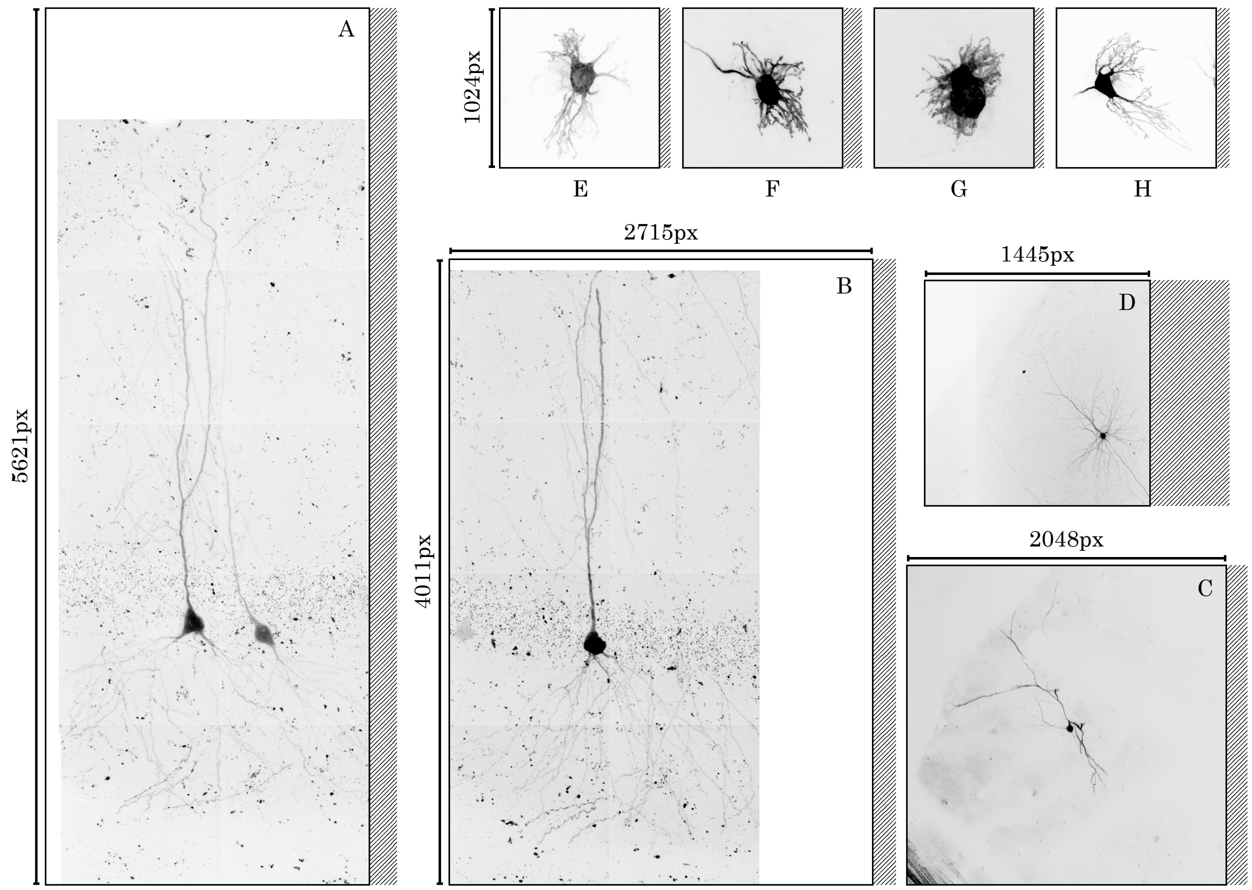

Figure S14: Examples of neurons that could not be reconstructed (A-C) or were very challenging to reconstruct correctly (D-H). For cases A-C only APP2 yielded reconstructions but with very low F-scores. Here the image stacks are visualized as maximum-intensity projections with inverted gray-levels together with their dimensions in pixels and scaled to retain their relative sizes for comparison. The width of the striped pattern on the right side of each image corresponds to the number of slices in the stack. Possible reasons why the methods failed on these cases could be the size of the image stacks, background structures, the complexity of the neurons, or poor fluorescent labeling.

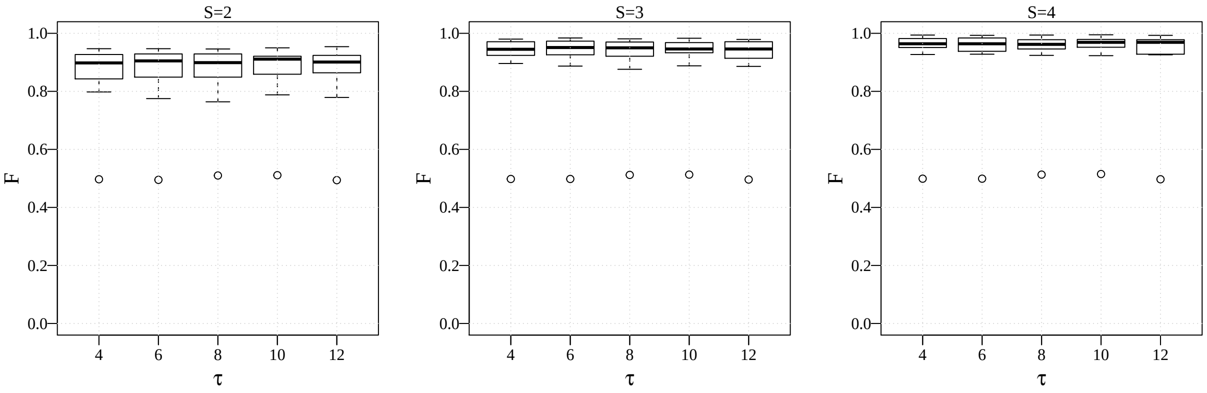

Figure S15: F scores of our PNR method as a function of the noise tolerance parameter τ for the OPF data set. Results are shown for S = 2 (left), 3 (middle), and 4 (right).

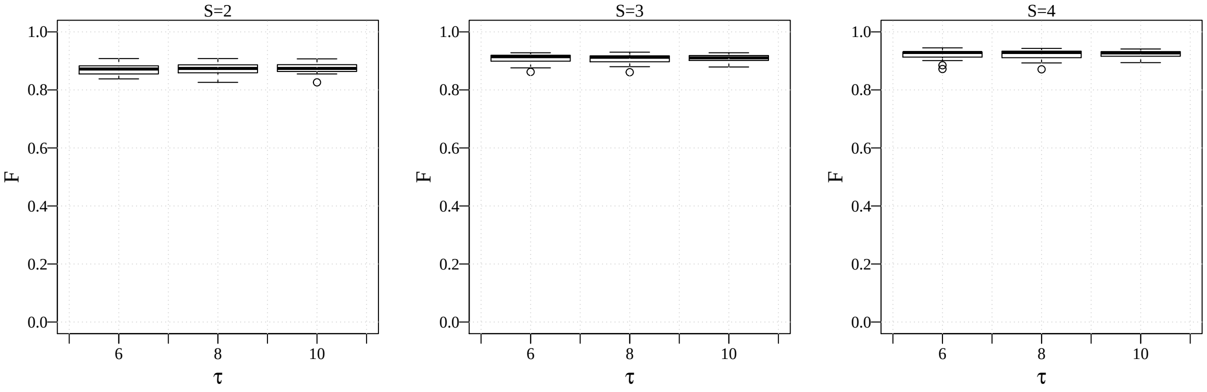

Figure S16: F scores of our PNR method as a function of the noise tolerance parameter τ for the NCL1A data set. Results are shown for S = 2 (left), 3 (middle), and 4 (right).

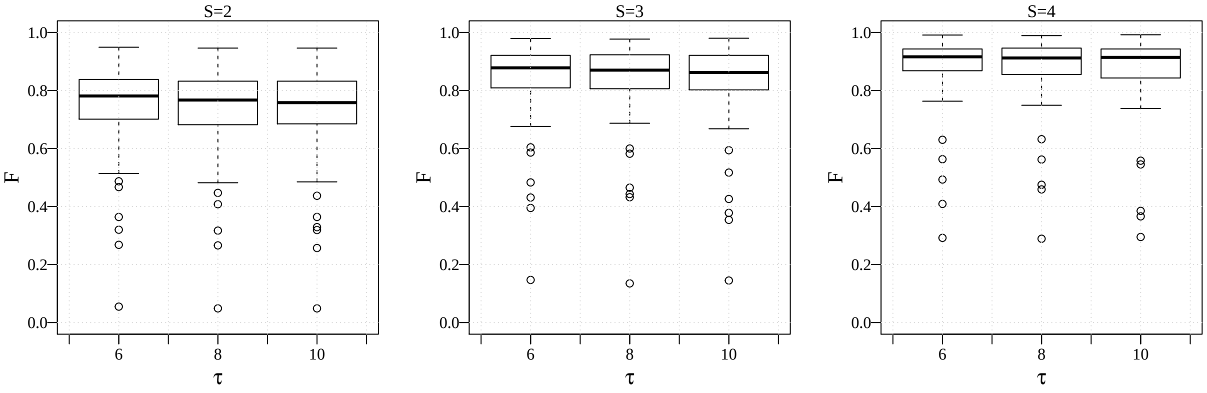

Figure S17: F scores of our PNR method as a function of the noise tolerance parameter τ for the BGN data set. Results are shown for S = 2 (left), 3 (middle), and 4 (right).

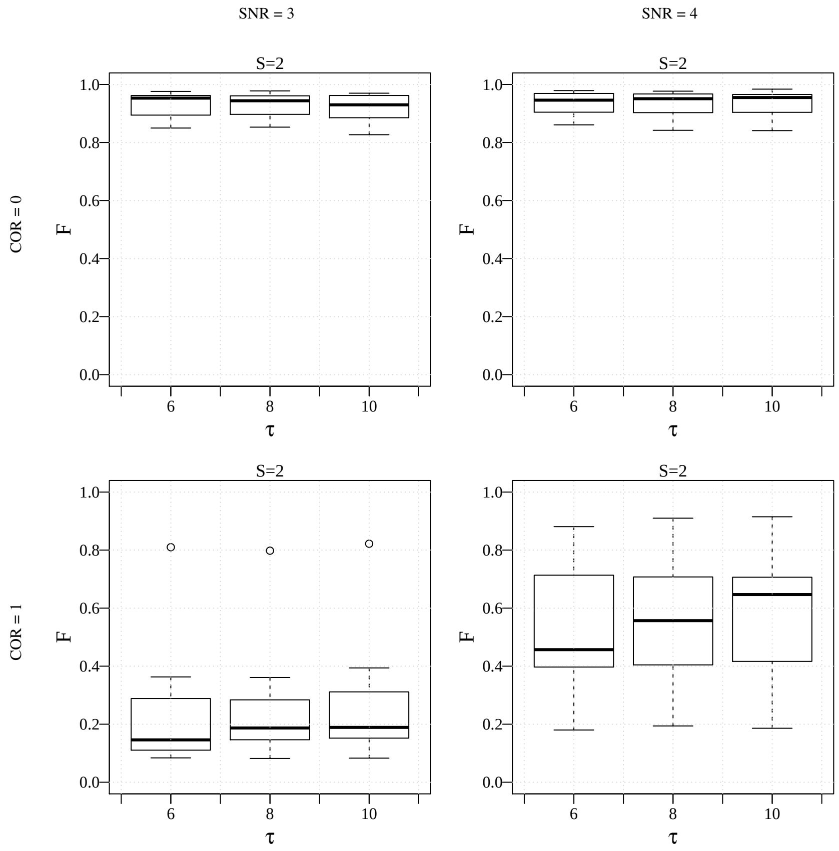

Figure S18: F scores of our PNR method as a function of the noise tolerance parameter τ for the synthetic images. Results are shown for S = 2 (all cases) and COR = 0 (top) and 1 (bottom) in combination with SNR = 3 (left) and 4 (right).

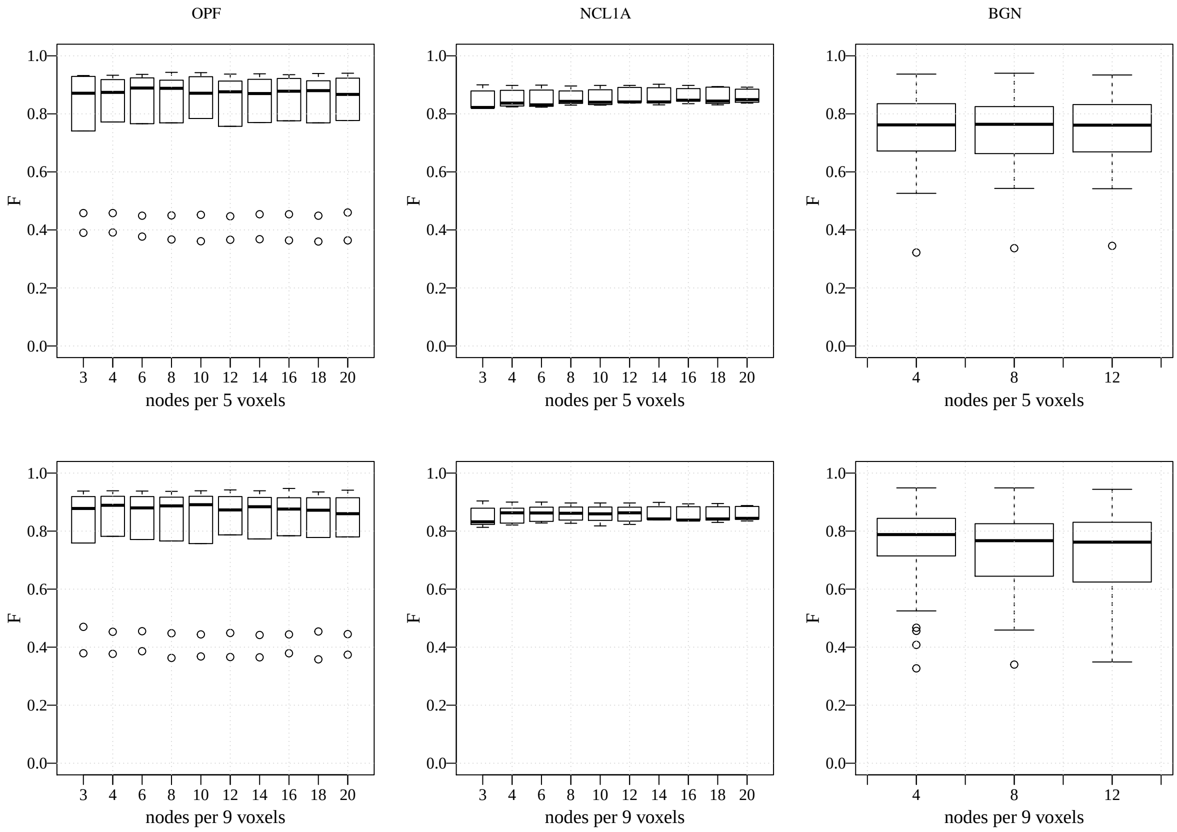

Figure S19: F scores of our PNR method as a function of the node density limit parameter δ_n for the different real data sets. Results are shown for $n = 5$ (top) and 9 (bottom) voxels and for the OPF (left), NCL1A (middle), and BGN (right) data sets.
